# Site specific N- and O-glycosylation mapping of the spike proteins of SARS-CoV-2 variants of concern

**DOI:** 10.1038/s41598-023-33088-0

**Published:** 2023-06-21

**Authors:** Asif Shajahan, Lauren E. Pepi, Bhoj Kumar, Nathan B. Murray, Parastoo Azadi

**Affiliations:** 1 Vaccine Production Program, Vaccine Research Center, National Institutes of Health, 9 W Watkins Mill Rd, Gaithersburg, MD 20877 USA; 2 Complex Carbohydrate Research Center, University of Georgia, 315 Riverbend Rd, Athens, GA 30602 USA

**Keywords:** Carbohydrates, Analytical chemistry, Analytical biochemistry, Mass spectrometry, Proteomic analysis, Structure determination

## Abstract

The glycosylation on the spike (S) protein of the severe acute respiratory syndrome coronavirus 2 (SARS-CoV-2), the virus that causes COVID-19, modulates the viral infection by altering conformational dynamics, receptor interaction and host immune responses. Several variants of concern (VOCs) of SARS-CoV-2 have evolved during the pandemic, and crucial mutations on the S protein of the virus have led to increased transmissibility and immune escape. In this study, we compare the site-specific glycosylation and overall glycomic profiles of the wild type Wuhan-Hu-1 strain (WT) S protein and five VOCs of SARS-CoV-2: Alpha, Beta, Gamma, Delta and Omicron. Interestingly, both N- and O-glycosylation sites on the S protein are highly conserved among the spike mutant variants, particularly at the sites on the receptor-binding domain (RBD). The conservation of glycosylation sites is noteworthy, as over 2 million SARS-CoV-2 S protein sequences have been reported with various amino acid mutations. Our detailed profiling of the glycosylation at each of the individual sites of the S protein across the variants revealed intriguing possible association of glycosylation pattern on the variants and their previously reported infectivity. While the sites are conserved, we observed changes in the N- and O-glycosylation profile across the variants. The newly emerged variants, which showed higher resistance to neutralizing antibodies and vaccines, displayed a decrease in the overall abundance of complex-type glycans with both fucosylation and sialylation and an increase in the oligomannose-type glycans across the sites. Among the variants, the glycosylation sites with significant changes in glycan profile were observed at both the *N*-terminal domain and RBD of S protein, with Omicron showing the highest deviation. The increase in oligomannose-type happens sequentially from Alpha through Delta. Interestingly, Omicron does not contain more oligomannose-type glycans compared to Delta but does contain more compared to the WT and other VOCs. O-glycosylation at the RBD showed lower occupancy in the VOCs in comparison to the WT. Our study on the sites and pattern of glycosylation on the SARS-CoV-2 S proteins across the VOCs may help to understand how the virus evolved to trick the host immune system. Our study also highlights how the SARS-CoV-2 virus has conserved both *N*- and *O*- glycosylation sites on the S protein of the most successful variants even after undergoing extensive mutations, suggesting a correlation between infectivity/ transmissibility and glycosylation.

## Introduction

In late 2019 severe acute respiratory syndrome coronavirus 2 (SARS-CoV-2) emerged and rapidly erupted into a global pandemic by March 2020. SARS-CoV-2 results in coronavirus disease 19 (COVID-19), which can result in fever, cough, and myalgia amongst other serious symptoms^1–6^. Scientists around the world quickly shifted their research efforts to determine how to stop SARS-CoV-2 infection. As of February 2023, there have been 672 million confirmed cases of COVID-19 and 6.85 million deaths worldwide^7^. In December 2020 the FDA issued the first emergency use authorization (EUA) of a vaccine for the prevention of COVID-19 (Pfizer-BioNTech) in the United States^8–10^. Although vaccines have been made available to all American adults as of April 2021, these vaccines were designed using the Spike (S) protein of the wild type Wuhan-Hu-1 SARS-CoV-2 (WT)^2,9,11–16^. Since the initial SARS-CoV-2 was identified, many variants have emerged. Of these variants, five (B.1.1.7 (Alpha), B.1.351 (Beta), P.1 (Gamma), B.1.617.2 (Delta), B.1.1.529 (Omicron)) were deemed variants of concern (VOCs), and multiple derivatives of B.1.1.529 have become the dominant VOC in recent months^17^. VOCs are classified by the World Health Organization (WHO) as having an increase in transmissibility or detrimental change in COVID-19 epidemiology, or an increase in virulence or change in clinical disease presentation, or a decrease in effectiveness of public health and social measures or available diagnostics, vaccines and therapeutics^17^. Research has shown the vaccines remain effective against these variants. In particular, the Pfizer-BioNTech vaccine is more than 95% effective against B.1.1.7 (Alpha) and B.1.351 (Beta) variants, and 88% effective against B.1.617.2 (Delta)^14,15^. A study from Israel showed that though the vaccine was still 90% effective against severe disease caused by the B.1.617.2 variant, it was only 39% effective against infection^14,18,19^. These findings explain the surge in infections noted worldwide due to B.1.617.2 despite high vaccination rates. Thus, the emergence of B.1.1.529 (Omicron) and its sub-lineages have highlighted the necessity to modify the current vaccines to better combat the new and emerging variants^20–23^.

B.1.1.7, also deemed the Alpha variant, was first designated in December 2020 in the United Kingdom^17^. This VOC contains a N501Y mutation which increases receptor-binding affinity of the spike protein with hACE2. As a result of this mutation, the B.1.1.7 variant is estimated to be 40–70% more transmissible and to have 30–50% increased mortality compared to the WT SARS-CoV-2^24^. The B.1.351 (Beta) variant was designated in South Africa at the same time as B.1.1.7^17^. This variant had a higher prevalence among young people with no existing health conditions. The P.1 variant (Gamma) was designated in Brazil in January 2021.^17^ This variant has an increase in both transmissibility and mortality compared to the native form. The B.1.617.2 (Delta) variant became a VOC in Spring 2021, though it had been monitored as a variant of interest (VOI) since December 2020^17^. The Delta variant has both increased transmissibility and mortality and a higher prevalence amongst young people than the WT virus. In July 2021 the Delta variant spread globally and became the dominant strain of SARS-CoV-2^17^. The B.1.1.529 (Omicron) variant was designated in late November 2021 and was the dominant strain of SARS-CoV-2 for about 6 months, until its descendants (BA.1–BA.5) emerged. Currently XBB.1.5, and Omicron subvariant, is the dominant SARS-CoV-2 strain^17,22^.

The SARS-CoV-2 S protein, which is a trimeric class I fusion protein, is used by the virus to bind to and enter human target cells^5,25^. The S protein is made of two subunits (S1 and S2). The S1 subunit achieves attachment of the virus to the host cell, and S2 subunit enables fusion of the SARS-CoV-2 and host cellular membranes^5^. The S protein of SARS-CoV-2 interacts directly with human angiotensin-converting enzyme 2 (hACE2) allowing infection of the host cell with SARS-CoV-2^3,26–30^. Due to the important role in the S protein for SARS-CoV-2 infection, it has been the target of vaccine candidates. The glycosylation of the WT S protein has been extensively studied over the past few years^1,2,4,5,25,31,32^. The WT S protein has 22 possible N-glycosylation sites, and all of these sites are fully or partially occupied^5,25^. The S protein also has 11 identified O-glycosylation sites^32^. However of these O-glycan sites, only site T323 has been identified consistently by multiple research groups. The glycans on the S protein allow for proper protein folding and priming by host proteases^1,5^. They are also believed to help evade innate and adaptive immune responses. Since the variants of concern have an increase in transmissibility and/or virulence, it stands to reason that there is a notable change in the S protein glycosylation.

To understand if these reported changes in transmissibility and virulence are related to S protein glycosylation patterns, we have performed site specific and global quantitative *N*-linked and *O*-linked glycan profiling of the S protein from WT, as well as VOCs B.1.1.7 (Alpha), B.1.351 (Beta), P.1 (Gamma), B.1.617.2 (Delta) and B.1.1.529 (Omicron). To accurately compare these S proteins, we have procured recombinant S proteins expressed in HEK293 cells. Multiple findings have been published on the glycosylation of the WT S protein as well as several publications on the variants^4,5,25,33,34^. Glycosylation differences are noted across these studies, with the most likely cause being differing expression systems and expression methods. Proline substitutions are utilized to stabilize the prefusion conformation of recombinant S protein. Research has shown that these proline substitutions result in differing site-specific glycosylation, however these glycosylation changes were determined to not impact serological activity^33^. A study looking at the glycosylation of S protein isolated from infectious virions found that the viral S protein closely resembles the recombinant trimeric S protein expressed in HEK293 cells^31^. This study showed that proline mutations to stabilize prefusion confirmation, as well as abolishing the furin cleavage site and preventing S1 shedding, which are seen in recombinant trimeric S protein, allow glycosylation to most closely resemble the viral S protein.

Our amino acid sequence analysis showed that SARS-CoV-2 virus conserved all 22 N-glycosylation sites on S protein across the VOCs except for the loss of the N17 site in the Delta variant and addition of the N20 and N188 sites in the Gamma variant. However, the variants displayed alteration in the glycan pattern across the sites of the S protein. Starting with the Alpha variant and progressing to the Omicron variant, a slight increase in oligomannose and decrease in highly complex glycoforms was observed globally. Although the site occupancy of O-glycosylation is lower, the virus preserved the O-glycosylation sites across the variants including the highly mutated Omicron variant.

## Materials and methods

Sequencing-grade modified trypsin (Cat No. V5111) and chymotrypsin (Cat No. V1061) were purchased from Promega (Madison, WI). The SARS-CoV-2 spike proteins were purchased from R&D systems (St. Paul, MN) (Cat. No’s 10561-CV-100, 10796-CV-100, 10786-CV-100, 10795-CV-100, 10878-CV-100, and 11060-CV-100). Dithiothreitol (DTT), iodoacetamide (IAA) and α-lytic protease (Cat No. A6362) were purchased from Sigma Aldrich (St. Louis, MO). All other reagents were purchased from Sigma Aldrich unless noted otherwise. Data analysis was done using Byonic 4.2 software and manually using Thermo Fisher FreeStyle 1.8 and Xcalibur 4.2.

### Protease digestion

20 µg of the purified protein samples were resuspended in 50 mM ammonium bicarbonate (ABC) and treated with 25 mM DTT at 50 °C for 45 min. To this, 90 mM IAA was added, and the samples were incubated at room temperature in the dark for 20 min. The samples were then passed through a 10-kDa Amicon Ultra centrifugal filter (cat no. UFC501096). Briefly, the samples were loaded onto the filter and centrifuged at 14 000×*g* for 15 min. 400 µL of 50 mM ABC was then added and centrifugation was repeated once. The concentrated samples were then resuspended once more in 50 mM ABC and treated with trypsin, chymotrypsin and/or *α*-lytic protease. Trypsin and α-lytic digests were incubated at 37 °C overnight. Chymotrypsin was incubated at room temperature overnight. The samples were then heated to 100 °C for 5 min to deactivate the proteases. For double digestions, the samples were re-treated with the indicated enzyme, and digestion was halted in the same manner. Before LC–MS analysis, the samples were filtered using 0.2-µm filters (Cat. No ODM02C34) and diluted in 0.1% formic acid (FA)^3,5,6^. Samples were analyzed in duplicate.

### ^18^O labeling of peptides

The proteins were digested with trypsin as outlined above. Following trypsin digestion, the peptides were cleaned using a C18 Solid Phase Extraction (SPE) cartridge and eluted with 1 mL each of 20%, 40% and 100% isopropanol in 5% acetic acid. The sample was then dried using a speed vacuum concentrator (SpeedVac™) and resuspended in 36 µL of ^18^O water. A 2 µL portion of 1 M sodium phosphate buffer (pH 6.8) was added, along with 2 µL of PNGase F. The sample was incubated at 37 °C overnight. Solvents were removed using the SpeedVac™. 50 µL of 50 mM ammonium bicarbonate was added to the dried sample with 2 µL of trypsin (0.5 µg/µL) and incubated at 37 °C for 6 h. The sample was then loaded onto a C18 SPE cartridge (Resprep, 26,030), and N-glycans were eluted with 5% acetic acid. Peptides were eluted with 1 mL each of 20%, 40% and 100% isopropanol in 5% acetic acid. The solvents were removed on the SpeedVac™. The peptides were then resuspended in 0.1% formic acid and analyzed by LC–MS/MS^35^.

### N- and O-glycan release

50 µg of each S protein was dissolved in 25 µL of 50 mM ammonium bicarbonate in duplicate. A 25 µL aliquot of 25 mM DTT was added, and the samples were incubated at 50 °C for 60 min. A portion of 25 µL of 90 mM IAA was then added, and the mixture was incubated at room temperature in the dark for 20 min. The samples were then desalted using 10-kDa Amicon Ultra spin filters following manufactures recommendations. After desalting, 2 µL of PNGase F was added to the samples and incubated at 37 °C overnight. The released *N*-glycans were separated from the *O*-glycoprotein portion using 10-kDa Amicon Ultra spin filters. The released *N*-glycans (filtrate) were pooled and loaded onto a C18 SPE cartridge and eluted with 5% acetic acid and lyophilized. The sample remaining in the filter containing the O-glycoprotein portion were collected, lyophilized, then subjected to reductive *β*-elimination. Briefly, 250 µL of 50 mM sodium hydroxide (NaOH) solution was added to the dried samples. The pH was checked to confirm basic conditions, and 19 mg sodium borohydride (NaBH_4_) in 250 µL 50 mM NaOH was added, the samples were vortexed and then incubated at 45 °C for 18 h. The samples were cooled to room temperature and neutralized by adding 10% acetic acid. The neutralized samples were loaded onto an ion exchange resin (DOWEX H+) and the glycans were eluted with 5% acetic acid. The flow-through was then loaded onto C18 SPE columns and eluted with 5% acetic acid and lyophilized. Borates present on the samples were removed by adding 500 µL of MeOH-acetic acid (9:1) and drying under a stream of nitrogen. This process was repeated five times^3,5,35,36^.

### Per-O-methylation

A dimethylsulfoxide (DMSO)/NaOH base was made according to the method by Anumula and Taylor^37^. To the dried samples, 200 µL of DMSO and 300 µL of the DMSO/NaOH base were added, followed by 100 µL of methyl iodide. The sample vial was vortexed, and then mixed using a shaker for 15 min. The reaction was quenched using 2 mL of LC–MS grade water, and excess methyl iodide was purged by bubbling the mixture under a stream of N_2_. Following the removal of excess methyl iodide, 2 mL of dichloromethane was added, and the solution was mixed vigorously for 30 s to extract the permethylated glycans. The organic layer was separated and dried under a stream of N_2_. The dried sample was then resuspended in 300 µL of 50:50 MeOH and H_2_O, and 10 µL was injected for LC–ESI–MS/MS analysis^35,36,38^.

### Data acquisition

Glycopeptides were analyzed on a Thermo Fisher Orbitrap Eclipse Tribrid mass spectrometer (MS) equipped with a nano-electrospray source and coupled to a Dionex Ultimate RSLCnano liquid chromatography system. Prepacked nano-LC columns (15 cm length, 75 µm internal diameter) filled with 3 µm C18 material (reverse phase) were used. A 180-min gradient was utilized, with 0.1% formic acid as solvent A (aqueous) and 20% water, 80% acetonitrile, 0.1% formic acid as solvent B (organic) as shown in Supp Table S2. Samples were analyzed in positive-ion mode. Precursor ion scans were acquired at a resolution of 120,000 in the Orbitrap analyzer, and precursors were selected at a timeframe of 3 s for MS/MS fragmentation in the Orbitrap analyzer at a resolution of 30,000. The MS/MS trigger threshold was set to 1000 counts and monoisotopic precursor selection was enabled. Charge state screening was enabled and precursors with a charge of + 1, or an unknown charge were excluded. A dynamic exclusion duration of 30 s was enabled^3,5,6^. All data was collected in duplicate on different days. MS/MS fragmentation was done using stepped higher-energy collision induced dissociation (HCD) product triggered collision induced dissociation (CID) (HCDpdCID) program.

*N*- and *O*-glycan samples were analyzed using a Thermo Fisher Orbitrap Fusion Tribrid MS system coupled to a Dionex Ultimate RSLCnano liquid chromatography system. Prepacked nano-LC columns (15 cm length, 75 µm internal diameter) filled with 3 µm C18 material (reverse phase) were used for chromatographic separation of the glycans. A 72-min gradient was utilized, with 98% water, 2% acetonitrile, 0.1% formic acid and 1 mM sodium acetate as solvent A (aqueous) and 20% water, 80% acetonitrile, 0.1% formic acid and 1 mM sodium acetate as solvent B (organic) as shown in Supp Table S3. Precursor ion scans were acquired at a resolution of 120,000 in the Orbitrap analyzer, and precursors at a time frame of 3 s were selected for subsequent MS/MS fragmentation in the Orbitrap analyzer at a resolution of 15,000. Precursors with an unknown charge state, or charge state of + 1 were excluded, and dynamic exclusion was enabled (30 s duration)^39^. MS/MS fragmentation was conducted with fixed CID (Collision Energy 40%). All data was collected in duplicate on different days. ESI spectra shown in the supplemental material was obtained by averaging the LC–MS chromatogram across the 72 min run and deconvoluting the spectra using Thermo Fisher Freestyle 1.8 software.

### Sialic acid linkage

Permethylated glycans were dissolved in 50:50 MeOH:H_2_O with 1 mM lithium carbonate. The samples were injected directly into a Thermo Fisher Orbitrap Fusion Tribrid Mass Spectrometer. MS^n^ experiments were then performed to isolate and fragment the penultimate galactose residue (*m/z* 211.1) adjacent to the sialic acids of N-glycans. Analysis was performed in the ion trap, using quadrupole isolation. Diagnostic fragment ions were then evaluated to determine linkage as outlined previously^40^.

### Data analysis

Tryptic, chymotryptic, *α*-lytic, combined tryptic/chymotryptic and combined tryptic/*α*-lytic digests of the spike proteins were searched against the FASTA sequences, which were provided by R&D systems. The data was analyzed using Byonic software with semi-specific cleavage enabled and choosing the appropriate cleavage sites for each protease. Oxidation of methionine, deamidation of asparagine and glutamine were used as common variable modifications and carbamidomethylation of cysteine as a fixed modification. For ^18^O analysis, an additional rare modification of + 2.988261 at asparagine was searched. Common mammalian *N*- and *O*-glycans were also used as rare variable modifications. The specific databases used were provided from the Byonic software (*N*-glycans: common 309 mammalian, O-glycans: 9 most common mammalian). Additional Byonic runs were conducted using a custom glycan database based on the glycoforms identified in glycomics data, however no additional matches were obtained. The databases used were chosen to ensure no low abundant glycans were missed. A precursor mass tolerance of 5 ppm was set, and a fragment mass tolerance of 10 ppm. A maximum of 2 missed cleavages was allowed, as well as 2 common modifications per peptide, and 1 rare modification per peptide. LC–MS/MS spectra for all samples were also manually interpreted using Thermo Fisher FreeStyle 1.8 software to identify glycan oxonium ions, neutral loss patterns and glycopeptide fragmentation^3,5,6,41^.

When manually confirming the results, close attention was paid to oxonium ions detected in each MS/MS spectra. Specifically, oxonium ions for Neu5Ac (m/z 292.1026 and 274.0921) were checked for all matches with more than one fucose, but no sialic acid to rule out false assignment as multiple fucoses. Relative abundance of the peptides was determined using area under the curve. The same peptide backbone for an individual site was used for glycoform abundance, to eliminate any changes in ionization. This was also used to confirm assignments, as all positively assigned peptides were searched for in the raw data and verified. Elution times of peptides were also verified (i.e. sialic acid containing peptides are expected to elute later than those without sialic acid). If multiple peaks arouse when extracting peptide masses, the MS was checked to ensure the peak arouse from the expected mass, and not an isotope with a similar mass. Due to the extent of the data produced from these proteins, all data was cross-checked internally.

For glycomics, LC–MS/MS data was analyzed using Thermo Fisher FreeStyle 1.8, GlycoWorkBench 2.0 and manual intepretation^42^.

### Results and discussion

**Figure 1:**
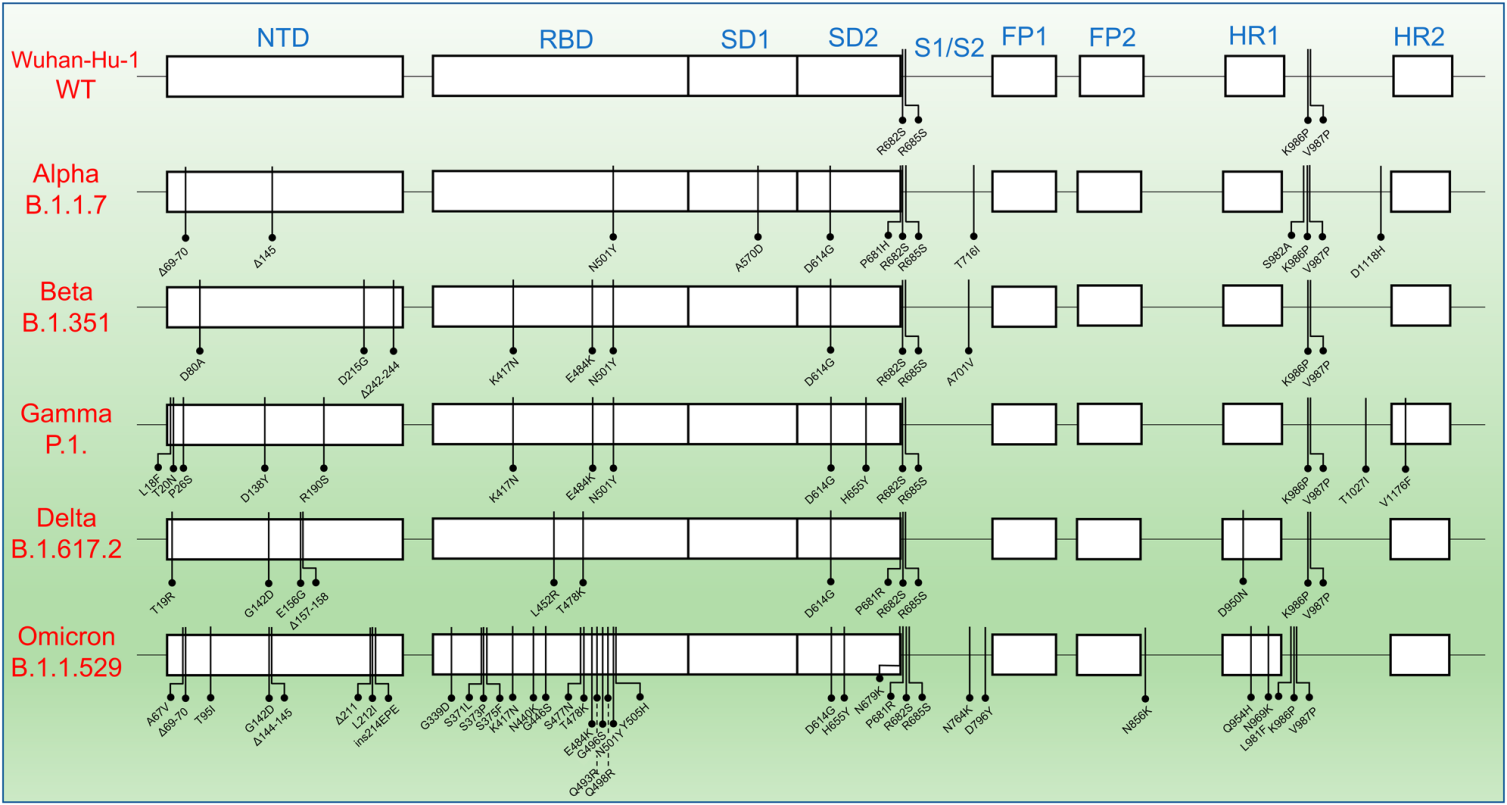
The mutations across the variants of concerns (VOCs) of SARS-CoV-2. Mutations shown represent the exact protein sequences used for this analysis, based on the sequence information provided by R&D systems. The mutations from the Wuhan-Hu-1 strain (WT) happened predominantly in the NTD and RBD domains and mutations increased significantly in the case of Omicron variant. NTD, N-terminal Domain; RBD, Receptor Binding Domain; SD1, subdomain 1; SD2, subdomain 2; FP1, Fusion Peptide 1; FP2, Fusion Peptide 2; HR1, Heptad repeat 1; HR2, Heptad repeat 2; S1, Subunit 1; S2, Subunit 2.

There are two WHO classes of SARS-CoV-2 variants, VOIs and VOCs. VOIs have mutations that improve SARS-CoV-2 transmissibility and immune evasion. VOCs are variants that have mutations that can lead to greater transmissibility and virulence as well as the ability to decrease the effectiveness of vaccines and treatments^43^. VOCs of SARS-CoV-2 have gained mutations on the S protein during the viral evolution, enhancing the viral transmissibility and immune system evasion. Certain mutations are shared between the VOCs, but unique mutations that provide characteristic advantages to the viruses are also observed among the VOCs (Fig. 1)^44^.

We examined the glycosylation profiles of recombinant S protein of the SARS-CoV-2 variants Alpha (B.1.1.7), Beta (B.1.351), Gamma (P.1), Delta (B.1.617.2) and Omicron (B.1.1.529) to the WT. Since glycosylation depends on both the expression system and protein conformation, variations in glycosylation of a single protein may differ when expressed in different cell lines. Since S protein expressed in HEK293 cells is believed to best resemble the S protein of the actual virus^45^, the recombinant S proteins of each variant used for this glycosylation profile study were purchased from R&D Systems (Fig. S1), as they have been expressed in HEK293F cells in the same manner.

We employed a strategy that includes multiple protease digestions to evaluate and compare the glycosylation at all N- and O-glycosylation sites of the S protein of all variants, and comparing the global *N*- and *O*-glycan profiles of variants through glycomics (Fig. S2)^1,5,25^. The site-specific glycan distribution and abundance of *N*-glycans on all 22 possible N-glycosylation sites (21 in the case of Delta variant, 24 in the case of Gamma variant) were characterized by glycoproteomics analysis using a combination of proteases including trypsin, chymotrypsin and *α*-lytic protease. The protease digests were directly analyzed by LC–MS/MS using HCD product-triggered CID tandem mass spectrometry. The LC–MS/MS data files were processed through Byonic software, and the glycoforms at each site of the S proteins of all six variants were determined. The glycopeptide annotation was conducted using a glycan database available with Byonic software and this annotation data matched with a subsequent search using a glycan database generated from the glycomics results. The glycopeptide spectra were evaluated manually for accuracy in the assignments and ambiguous spectra were eliminated. Subsequently, the glycoforms at each individual sites of the S proteins of each variant were quantified by evaluating the precursor MS1 peak area of each glycopeptide (regardless of Byonic annotation). The relative abundances of each individual glycoform were calculated manually through Xcalibur and FreeStyle software after spectral deconvolution.

For the characterization of the O-linked glycosylation sites, the recombinant S proteins were digested by chymotrypsin followed by trypsin and treated with PNGase F for *N*-deglycosylation (Fig. S2). The resulting O-glycopeptide peptide pools were separated from the released *N*-glycans by C18 SPE and analyzed by LC–MS/MS. The data were further processed by Byonic for *O*-glycopeptide identification and quantified manually by extracting the peak area of respective *O*-glycopeptides after the deconvolution of LC–MS/MS spectra.

### Highly conserved N-and O-glycosylation sites on S protein across the VOCs

**Figure 2:**
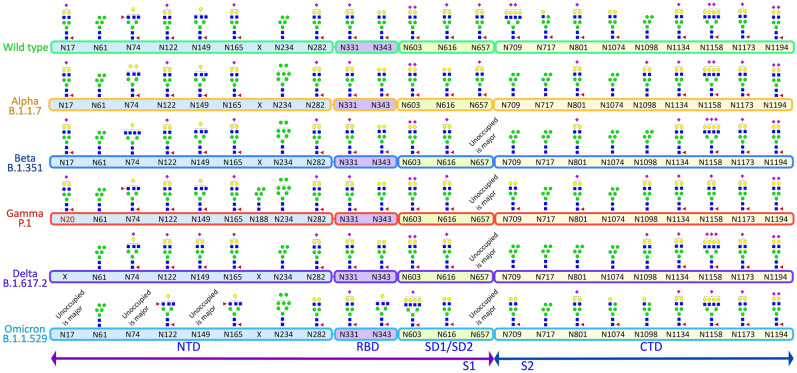
Most abundant glycan structure on each site of the WT and VOCs. Glycosylation sites are conserved in all the variants except for the presence of an additional site N20 in Gamma variant and loss of N17 in Delta variant. Most abundant glycoforms are similar across sites (examples being N61, N331, N1134, N1173 and N1194).

Presently a total of 3,033,576 human SARS-CoV-2 spike protein sequences, compromising from 1,270 to 1,273 amino acid residues, are available in the NCBI Virus database (https://www.ncbi.nlm.nih.gov/labs/virus, as of October 04, 2022). A previous study on the extent of mutation on the S protein from 303,250 human SARS-CoV-2 spike protein sequences revealed a total of 1,269,629 mutations at 1,229 distinct sites. This study observed ~ 96.54% mutations on the human SARS-CoV-2 spike protein sequence since the outbreak of the COVID-19 pandemic^46^. By analyzing 57,311 S protein sequences from the NCBI database, another study demonstrated that the N-glycosylation sites of SARS-CoV-2 S protein are highly conserved^47^. The glycosylation sites in the receptor binding domain (RBD), N331 and N343 and the surface glycosylation sites (N17, N149, and N657) were shown to have more than 99.67% shared identity. Our alignment of N-glycosylation sites on the VOCs revealed that all 22 N-glycosylation sites are precisely conserved across the variants except for the absence of site N17 on the Delta variant and presence of a new N20 site on Gamma variant (Fig. 2).

### Alpha (B.1.1.7) variant of SARS-CoV-2

Our glycoproteomic analysis shows that all 22 N-glycosylation sites and the T323 O-glycosylation site in the Alpha variant are occupied. Sites N122, N717, and N1098 showed increased sialic acid, while sites N74, N165, N282, N616, and N709 showed reduced sialic acid in the Alpha variant relative to WT. Oligomannose structures were elevated at sites N61, N234, N709, and N801 and reduced on site N1098 compared to WT. Sites N61, N709, N801, N1074 displayed reduced fucosylation, while only N717 showed an increase in fucosylation in the Alpha variant compared to the WT (Figs. 2, 3, 4, S3–S24).

**Figure 3:**
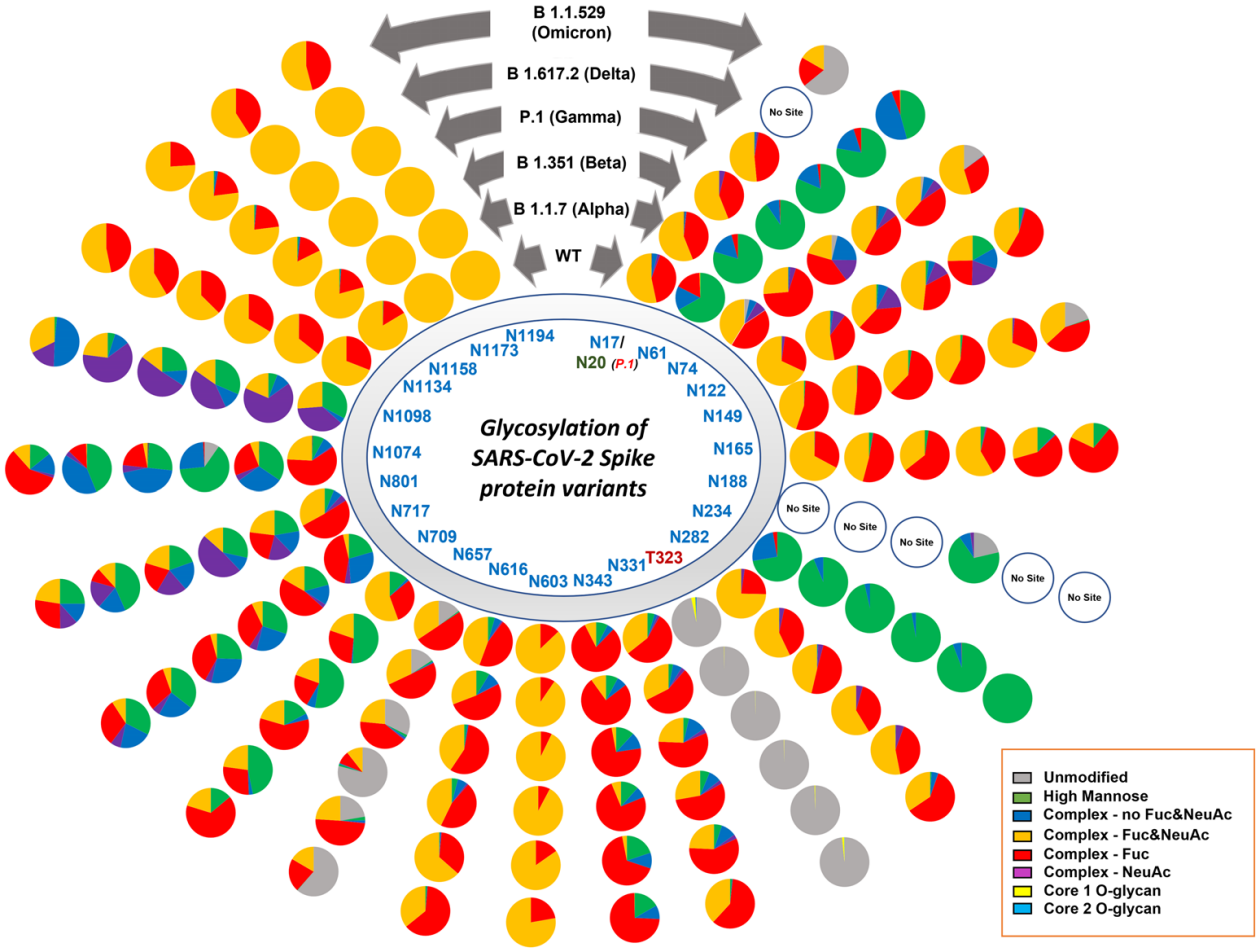
Site specific N- and O- glycans on SARS-CoV-2 spike protein across the WT and VOCs. Each pie chart denotes glycosylation sites of the S protein for each variant and shows the distribution of N- and O-glycosylation types at each site of the WT and VOCs.

**Figure 4:**
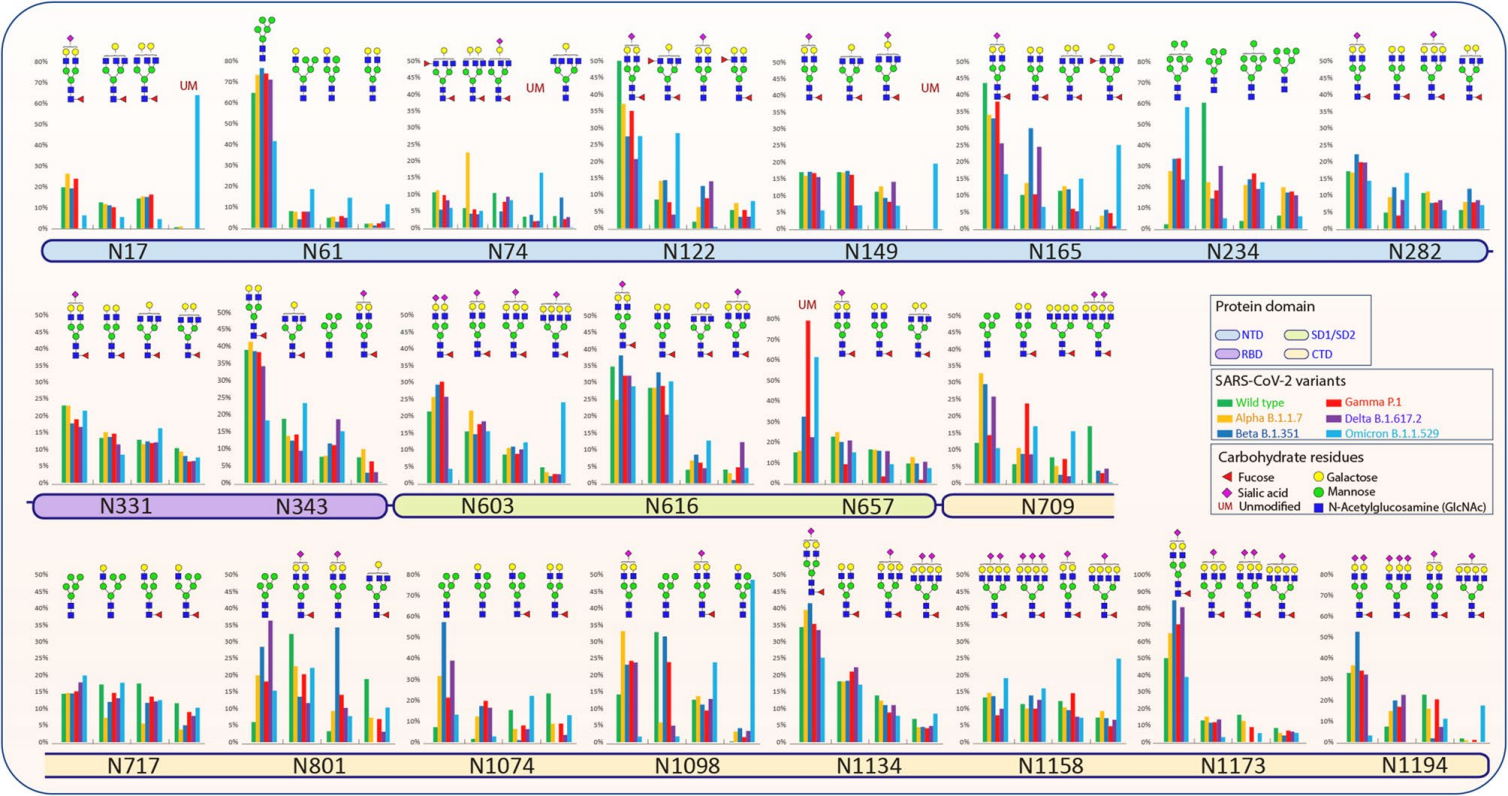
Relative abundances of four most intense glycoforms at each site of WT and VOCs.

The O-glycosylation at T323 in the Alpha variant showed predominantly core-1 glycans, while WT contains both core-1 and core-2 glycans. The overall occupancy of O-glycans in site T323 is lower in Alpha. This lower occupancy may be responsible for the reduced detection of low abundance core-2 structures (Figs. 3, S25), rather than a different ratio of core-1 and core-2 glycans.

### Beta (B.1.351) variant of SARS-CoV-2

A site-specific glycosylation comparison of Beta to the WT showed increased sialic acid at sites N603 and N801 but reduced sialic acid at sites N165, N282, N331, N709, and N1074. Comparison of oligomannose between Beta and WT showed increase at sites N61, N234, N343, N709, N717, N801, and N1074 for the Beta variant. Fucosylation also showed an increase at sites N61, N74, N122, N657, N709, N717, N801, N1074, and N1098 in comparison to WT (Figs. 2, 3, 4, S3–S24).

Following the trend of Alpha variant, glycoproteomics analysis showed the presence of only core-1 O-glycans and lower site occupancy at site T323 of Beta with respect to WT (Figs. 3, S25).

### Gamma (P.1) variant of SARS-CoV-2

Interestingly, the mutation T20N (replacing a threonine with an asparagine) and R190S (replacing an arginine with a serine) introduced two new N-glycosylation sites in the spike of Gamma variant, which could alter the viral shielding and antibody protection^44^.

**Figure 5:**
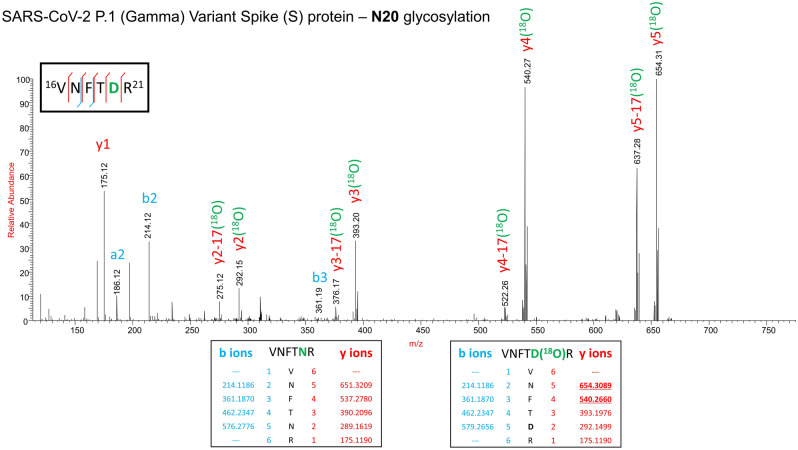
Confirmation of the presence of N-glycosylation at N20 site and absence of N-glycosylation at N17 site on the spike protein of the Gamma variant by stable ^18^O isotope labelling technique. HCD MS/MS spectrum of the peptide VNFTNR showing b and y fragment ion series, which confirms the ^18^O label is only at N20 (MS1, y2, y3, y4, y5 with 2.98 Da mass) thereby confirming the presence of N-glycosylation. Inset: in silico predicted b and y ion masses of peptide VNFTNR without deamidation or ^18^O label (left) and with ^18^O label (right)*.*There was no deamidation or ^18^O label noted on N17.

Since the Gamma variant contains an additional N-glycosylation site (N20) proximal to N17, we performed ^18^O labelling to confirm the site occupancy at N17 and N20. Interestingly, we obtained clear evidence for the presence of N-glycosylation on the newly introduced site in Gamma, but no glycosylation at the original N17 site (Fig. 5). This is supported by a recent publication by Newby et al., where site N17 was determined to be unoccupied, and site N20 occupied with N-glycans^34^.

The additional N-glycosylation site N188 is not proximal to other N-glycosites. Our experiments show this site is occupied with N-glycans, with majority of the occupancy being oligomannose type. We also found a large portion of this site (~ 20%) to be unoccupied. Interestingly, Newby et al. also found this site to be occupied with mainly oligomannose type glycans^34^.

Site-specific glycosylation showed the presence of similar types of N-glycans at site N20 in Gamma in comparison to the N-glycans at N17 of WT. Increased sialic acid was observed at site N1134 and reduced sialic acid at sites N282, N709, N1074 of Gamma in comparison to WT were observed. Like Beta, Gamma also showed only increased oligomannose at sites N61, N234, N343, N801, and N1074 with respect to WT. Fucosylation also showed reduction at sites N61, N122, N801, N1074, N1098 in Gamma with no sites showing an increase in Fucosylation in comparison to WT. Moreover, we observed an increase in unoccupied peptides at site N657 with respect to WT (Figs. 2, 3, 4, S3–S24). The Gamma variant also showed lower occupancy of core-1 type glycans at O-glycan site T323 (Figs. 3, S25).

### Delta (B.1.617.2) variant of SARS-CoV-2

Interestingly, our glycoproteomic study on the S protein of the Delta variant showed N-glycosylation occupancy at all sites except for N17 due to loss of the consensus sequence. A comparison of sites with significant differences in Delta to WT showed increased sialic acid at sites N149, N616, and N1098 but reduced sialic acid at sites N165, N282, N331, N657, N709, N1074, and N1134. Regarding oligomannose type glycans, increases were observed at sites N61, N122, N165, N234, N343, N709, N717, N801, and N1074 whereas site N1098 had decreased abundance. Reduced fucosylation was observed in Delta in comparison to WT at sites N61, N122, N717, N801, and N1074. Unoccupancy at site N657 was observed in Delta but not in the WT (Figs. 2, 3, 4, S3–S24). Like earlier mutant variants, the Delta variant also showed lower occupancy of core-1 O-glycans at site T323 in comparison to WT (Figs. 3, S25).

### Omicron (B.1.1.529) variant of SARS-CoV-2

The Omicron variant is the most mutated variant among all previous VOCs. Omicron also displayed the highest variation in glycan occupancy and glycan profile, but interestingly preserved all N- and O-glycosylation sites in the same manner as the WT. Most N-glycosylation sites showed significant differences on Omicron in comparison to WT. Our glycoproteomic study on the Omicron variant S protein showed increased sialic acid only at site N74, but reduced sialic acid were observed at sites N17, N122, N149, N165, N282, N343, N603, N709, N1134, N1173, and N1194, compared to the original WT strain. Oligomannose in the Omicron S protein showed increases at sites N165, N234, N343, N717, N801, and N1074 and reduction in oligomannose at sites N61, N1098 with respect to WT. The Omicron variant showed reduction in fucosylation at sites N17, N61, and N801 in comparison to WT, which follows the same trend as all previous VOCs. Most significant differences in the Omicron variant with respect to the previous variants are increased unoccupancy at sites N17, N74, N149, and N657 (Figs. 2, 3, 4, S3–S24).

Interestingly, the O-glycosylation at site T323 in Omicron variant spike showed higher occupancy in comparison to other mutants but similar occupancy to that of WT. Both core-1 and core-2 type of glycans were observed (Figs. 3, S25). We ould not detect O-glycosylation at site T678.

**Figure 6:**
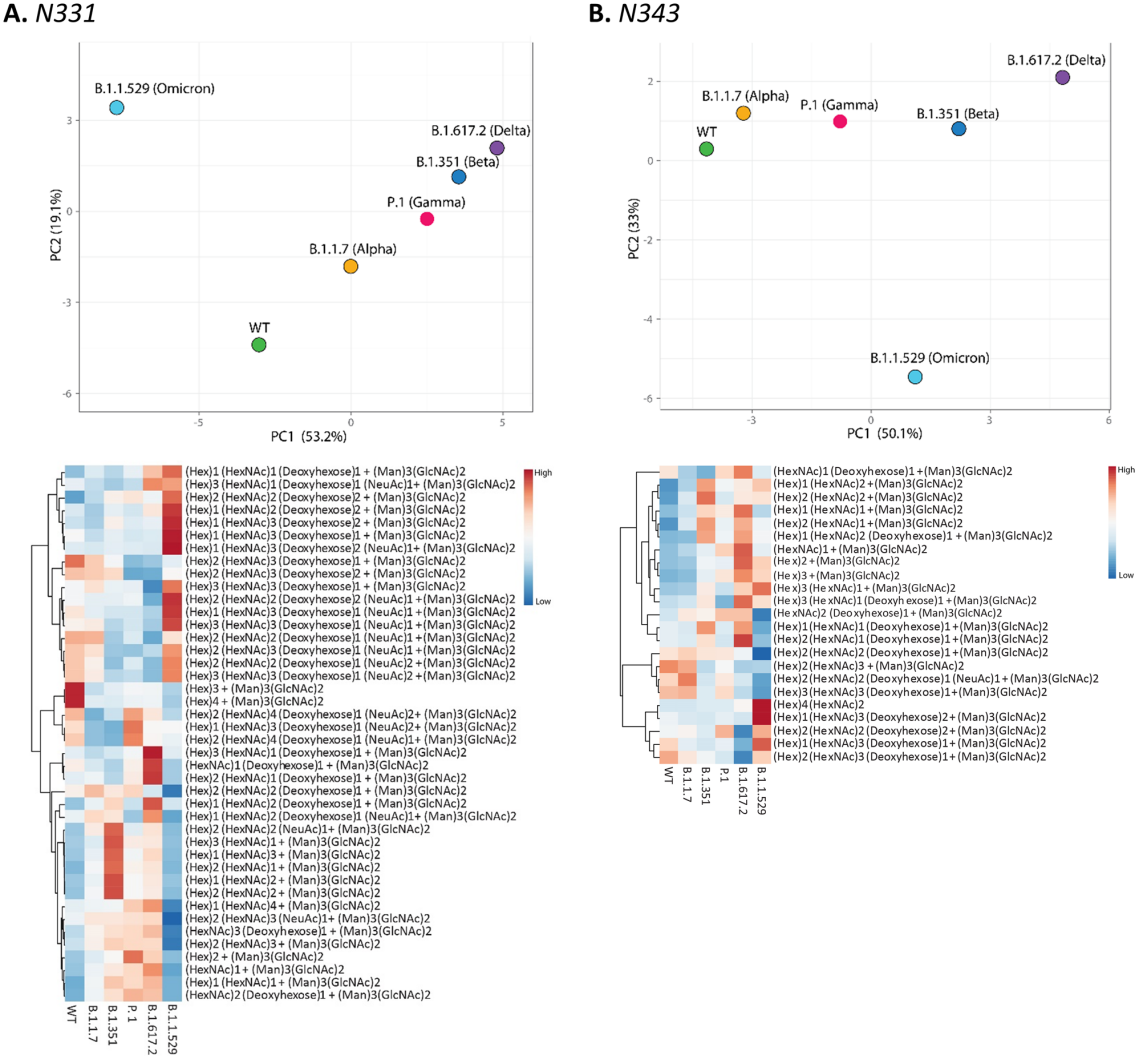
PCA analysis and heat map distribution of the N-linked glycans on sites at RBD, (**A**) N331 and (**B**) N343 showed distinct differences in the glycosylation on the VOCs in comparison to the WT, particularly the Omicron variant.

Site specific glycosylation comparison of the four most abundant glycoforms across the variants showed that NeuAc_1_Fuc_1_Gal_2_Man_3_GlcNAc_4_ and Man_5_GlcNAc_2_ were observed at the highest number of glycosylation sites for all samples compared to other glycoforms (Fig. 4). NeuAc_1_Fuc_1_Gal_2_Man_3_GlcNAc_4_ was detected as one of the four most abundant glycoforms in 15 of the 22 sites and Man_5_GlcNAc_2_ was detected as one of the top four most abundant glycoforms in 8 of the 22 sites. Comparison of site-specific quantitative N-glycosylation at each site of the spike protein across the variants by PCA analysis and heat map showed distinct variation in the VOC glycosylation in comparison to WT, particularly for the Omicron variant (Figs. 6, S26–S36).

### Distribution of overall N- and O-glycosylation on the S protein of VOCs: glycomics

**Figure 7:**
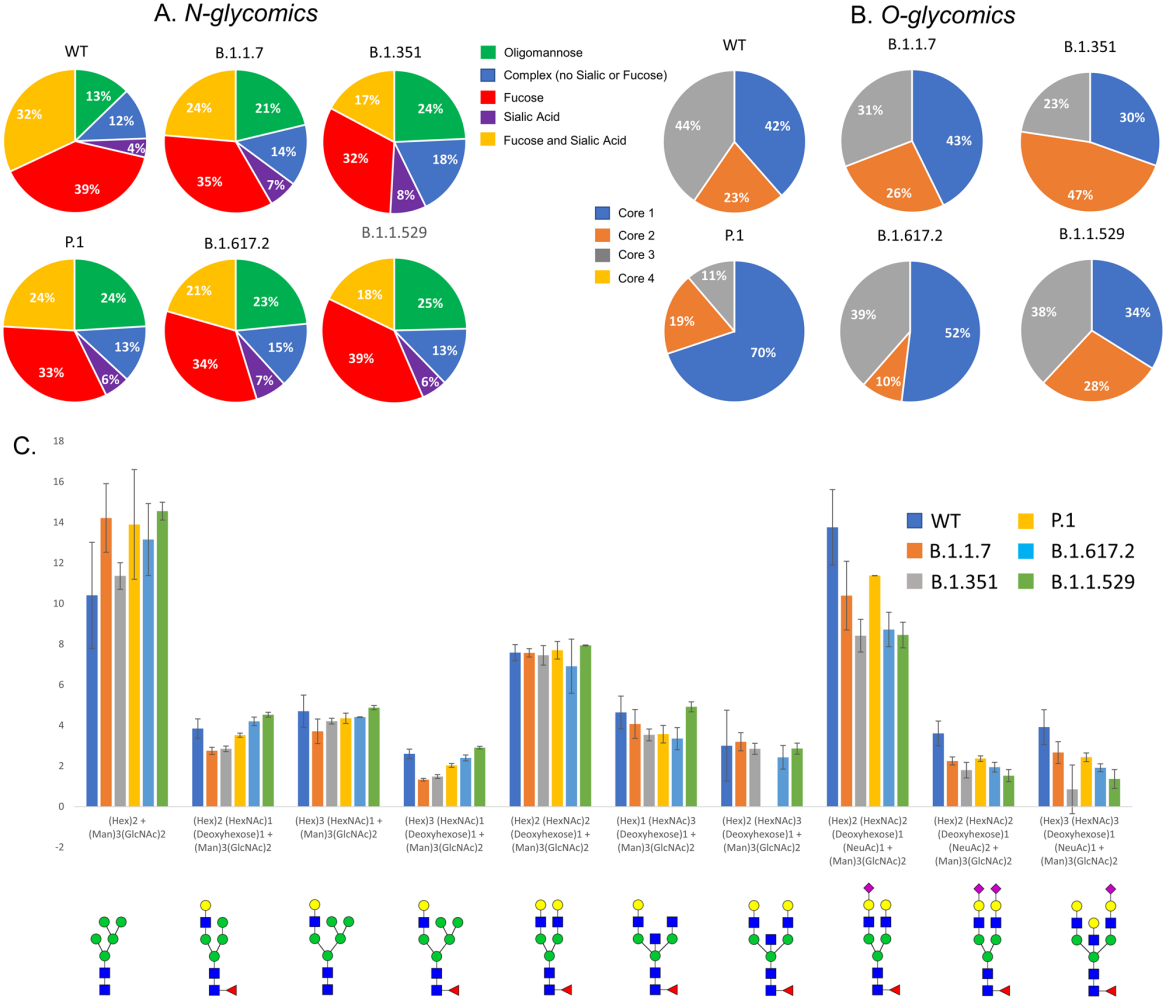
Distribution of (**A**) N- and (**B**) O- glycans among the VOCs determined by glycomics (released glycans). All variants showed differences in the type of N-glycans with respect to WT. (**C**). Relative abundance comparison of most abundant ten glycoforms of WT and VOCs. Omicron showed most significant overall N-glycan differences with 43% high mannose structures. Error bars represent standard deviation from duplicate analysis.

N-glycomic analysis of the S proteins of VOCs showed most significant differences in the WT and Omicron samples compared to the other four variants tested. The WT contains 13% oligomannose type glycans compared to other glycan types. The Alpha (21%), Beta (24%), Gamma (24%), Delta (23%) and Omicron (25%) variants all have a greater relative abundance of oligomannose than the WT (Figs. S37–S43). The site-specific analysis shows an increase in oligomannose for the VOCs at multiple sites compared to the WT S protein (Fig. 7).

We also determined the sialic acid linkages of the N-glycans isolated from WT and Omicron and observed the presence of both 2,3 and 2,6 linked sialic acids. Interestingly, even though the same cell lines were used for the expression of these proteins, Omicron showed more 2,6 linked sialic acids (Fig. S44, Table S1).

O-glycosylation of the spike variants is minor, but we identified core 1 O-glycans in all VOCs’ S proteins along with WT. WT and Omicron S proteins showed the highest O-glycan occupancy and presence of both core- 1 and core 2 glycans (Fig. 7).

### Changes in the oligomannose structures across the variants

The N-glycans at N331 and N343 located in the RBD have been reported mainly as complex types suggesting relatively better accessibility for the glycan processing^48^. We observed about 1–6% oligomannose structures at site N331 across the S proteins. While WT S protein displayed 7.6% oligomannose, subsequent variants displayed increasing levels: 7.9% on Alpha, 11.9% on Beta, 11.6% on Gamma, 19.9% on Delta and 16.8% on Omicron. Delta and Omicron, which are the most highly infectious variants among these, possessed relatively fewer fucosylated and sialylated glycans. The Delta variant obtained novel T478K and L452R mutations within the RBD which led to increased binding affinity to human ACE2 in comparison to other variants of concern^49,50^.

Delta and Omicron variants, which showed relatively higher oligomannose structures, escape from most of the antibodies isolated from convalescent patient sera, infected with early WT strain, and a reduction in neutralization by vaccine-elicited sera. Our results also showed that the Beta variant expressed slightly higher levels of oligomannose glycans at site N343 in comparison to Gamma variant (Fig. 3). Following the same trend, the Gamma variant is observed to be less resistant to naturally acquired or vaccine-induced antibodies than the Beta variant^13,51^. Agreeing with this observation, WT and the Alpha variant showed minimal differences in the sensitivity to several potently neutralizing antibodies^12^. Further experiments, including molecular modeling, would be needed to confirm these observations.

An increase in oligomannose structures was also observed at sites N61, N122, N165, N616, N801, and N1074 across all tested variants. Elevated oligomannose levels at certain sites of the S protein were observed in an earlier comparative study of Alpha variant with the WT^45^. The reduction in sialic acid and fucosylation across the variants are due to overall reduction in the complex-type glycans and corresponding increase in oligomannose structures (Fig. 3).

Our glycosylation profiling revealed that the Delta and Omicron variants showed more changes in surface glycan patterns, which is intriguing as they also have increased resistance towards vaccinated and naturally acquired host immune responses. Influence of these altered glycans on the conformation of the RBD of S protein and its interaction with ACE2 is not yet known. Molecular modeling of the interaction of RBD with ACE2 under altered glycan attachments could shed light on such factors.

### O-glycosylation sites are conserved across VOCs

O-glycosylation is involved in protein function and stability. Viral O-glycosylation is believed to play a role in the biological activity of viral proteins^5^. Past analyses have shown the SARS-CoV-2 WT S protein is O-glycosylated up to 11 sites, including T323 and T678^4,5,32,52,53^. It is important to note that the majority of these analyses did not use the same recombinant S protein. The expression system used for the SARS-CoV-2 structural proteins has been shown to be imperative for reproducible results^6^. Additionally, when expressing recombinant S proteins, mutations at the S1/S2 polybasic cleavage sites are introduced to stabilize the trimeric spike protein. Variations at these sites which are closer to the T678 O-glycosylation site could possibly affect the glycosylation profile at this site^54^.

O-glycosylation at T323 has been unambiguously detected by researchers who profiled glycosylation on the S protein of SARS-CoV-2, including the viral particles isolated from the infected patients^5,31,32^. Incidentally, all variants carried sialylated O-glycans at site T323, albeit at lower levels. The WT showed the highest glycan site occupancy (3.38%) (Figs. 3, S25), followed by the Omicron variant (1.62%). Interestingly, the Alpha, Beta, Gamma, and Delta variants showed similar unoccupied peptide levels at site T323 (99.8%, 99.7%, 99.8%, and 99.5%, respectively), with 0.5% or less site occupancy. These observations suggest that the accessibility of T323 within the RBD domain towards the O-glycosylation machinery is susceptible to conformational changes of the S proteins due to mutations occurring elsewhere on the protein. The conservation of site T323 following as many as 30 (in the case of Omicron) S protein mutations may be indicative of the relevance of O-glycosylation at this position. Though the site has very low site occupancy, it is curious that not only has the site been conserved in the most successful variants of SARS-CoV-2 (i.e., WT and VOCs), but the glycosylation at the site as well. This data suggests low levels of O-glycosylation may play an important role in viral binding to the host; however, the low site occupancy possibly suggests a lower impact of the O-glycans on the function of the S protein compared to the N-glycans within the RBD. Moreover, this suggests that a pan-SARS-CoV-2 vaccine could be developed targeting the O-glycosylation site of S protein.

Interestingly, it has been reported that mutations near the furin cleavage site at P681 of the Alpha and Delta variants lead to a decrease in O-glycosylation at site T678 and increased furin cleavage and syncytia formation^52^. This mutation also exists in the Omicron variant. However, we could not detect O-glycosylation at T678, possibly due to its lower occupancy or lack of expression on the S protein samples we studied. The recombinant S proteins used for our analysis contain R682S and R685S mutations which may inadvertently affect glycosylation. We were also unable to detect O-glycosylation at the other previously reported O-glycosites. This could also be due to lower site occupancy or lack of expression. A similar finding was reported previously when comparing S protein from viral particles to recombinant trimeric S protein. The viral S protein was found to have O-glycosylation at T678, but the recombinant protein tested was determined to not contain glycosylation at this site^31^.

Currently the roles of O-glycosylation on viral protein are not well known. Sialylated O-glycans have been shown to mediate binding of HSV-1 gB to cellular receptors on immune cells, so it is possible the consistent identification of sialylated O-glycans on the S proteins of the VOCs is allowing the virus to mediate hACE2 binding^55–57^. Other studies have shown evidence for a more general importance of O-glycosylation in viral binding, with mutation of O-glycosites leading to a decreased affinity for viral-immune cell binding^55,58^. This observation may explain the increase in O-glycosite occupancy in the Omicron variant at T323 compared to the other VOCs, as Omicron has an increased transmissibility compared to the other VOCs. Mutation studies and glycosylation evaluation on viral particles from patient samples would be necessary to confirm these theories.

## Conclusions

Glycosylation of the spike (S) protein of SARS-CoV-2 Wuhan-Hu-1 wild type strain (WT) has been extensively characterized^4,5,25^. Initial work highlighted the importance of specific glycan epitopes for binding of the S protein with hACE2^59^. Each VOC was characterized based on its increased transmissibility and mortality. Due to the importance of glycosylation for the binding to hACE2, we hypothesized that glycosylation changes may be causing the increase in transmissibility of these variants compared to the WT strain. Comprehensive N- and O-glycan analysis showed that all glycosites are conserved across these variants, with the exception of N17 in the B.1.617.2 (Delta) variant and addition of N20 and N188 in the P.1 (Gamma) variant. Multiple studies have shown that N-glycosylation sites are highly conserved across many of these S protein sequences. We believe this highlights the importance of glycosylation for SARS-CoV-2 survival and transmission, as all current VOCs have highly conserved glycosylation sites, aside from the Delta variant which lacks site N17 due to a T19R mutation. Interestingly, we found evidence of the new N20 glycosite in the Gamma variant being glycosylated but could not detect glycosylation on the original glycosite N17. This was determined both by fragmentation of the peptide, as well as ^18^O labeling of N-glycosylation sites. It appears that the glycosylation machinery “mistakes” the N20 site for the N17 site as these two sites are in close proximity to one another. This is further supported by the glycosylation profile at N20 of Gamma being like that of N17 of the other variants. Additionally, an R190S mutation in the Gamma variant resulted in the addition of a unique N-glycosite, N188, a site absent from the other five samples analyzed. This site was determined to be occupied with mainly oligomannose type glycans (~ 68%).Though glycosylation was conserved across the glycosites, changes in the glycoforms present at each site were noted for all S proteins tested.

The overall extent of complex glycans decreased sequentially from Alpha through Delta compared to the WT S protein. The Omicron variant also displays a decrease in complex-type compared to the WT, however the Omicron variant does not follow the same sequential decrease as the other variants. Interestingly, the glycoproteomics data indicates the Delta variant having the highest amount of oligomannose type glycans (Figs. 3, S2–S24); however, glycomics data suggests that all VOCs have elevated oligomannose glycans ranging from 20 to 26% compared to WT, which has 13% (Fig. 7). The Omicron variant has an increase in the unmodified peptides (i.e. the peptide with no glycosylation) at sites N17, N74, N249, and N657, which all possess mainly complex glycoforms. When comparing these findings to other published research on the S protein of WT SARS-CoV-2 and VOCs, similar trends are observed. Kuo et al.^45^ compared the WT to the Alpha variant via glycoproteomic analysis and found similar trends in N-glycan classes identified at each N-glycosite. A few notable differences are seen though, including sites N165, N603, N1074 were reported to contain mainly oligomannose structures by Kuo et al., whereas our findings identified a high abundance of complex/ hybrid structures at these sites. A recent publication by Newby et al. compared the WT S protein to the Beta, Gamma, Delta and Omicron variants^34^. Newby et al. also identified the two new N-glycosites in the Gamma variant, and also concluded that site N17 is not glycosylated, whereas N20 is instead. There were some differences noted in the class of glycans at specific sites, including N603 which we identified as containing no oligomannose, Newby et al. identified this site as being mainly oligomannose. In the case of both papers mentioned, all studies utilized S protein expressed in HEK293F cells. The major difference in the sample types is that Newby et al. and Kuo et al. both expressed S protein themselves, whereas for this study we purchased the purified glycoprotein^34,45^. It is possible that different protein tags and additional mutations used in the expression of the proteins could contribute to these differences in glycosylation noted between the studies. A recent publication by Xie et al.^60^ compared the WT S protein glycosylation to that of the Alpha, Beta, Gamma, Kappa, Delta and Omicron variants. This study focused on released glycans and did not look at site-specific changes. Overall, this study noted varying abundances of specific N-glycans and glycan classes from sample to sample, which we noted as well. Similar to our analysis, this study found Omicron to be the most different from the WT and other VOCs based on PCA analysis^60^.

The increased transmissibility of the VOCs reported so far correlates with the changes in glycosylation patterns noted in this manuscript. The WT S protein has less oligomannose-type and more complex-type glycans compared to the 5 VOCs examined. The Delta and Omicron variants, which have the highest resistance to natural and vaccine-induced antibodies, have the most significant increase in oligomannose-type compared to the WT.

The WT had more O-glycosylation compared to the 5 VOCs, however all samples possessed O-glycosylation at site T323, with predominantly core-1 O-glycans (Figs. 3, S25). Previous reports have shown the WT S protein as being heavily O-glycosylated with up to 11 O-glycosites^32^. Our glycoproteomic analysis was unable to confirm this, and we were only able to identify 1 O-glycosite (T323). This is likely due to the specific expression system and method used to produce the recombinant S protein. Many past studies have shown the importance of the expression system and how this affects the glycosylation patterns^6^. This is likely the reason for various glycosylation studies of the S protein yielding differing results. In addition to the expression system, any additional mutations introduced to the protein during expression can affect availability of glycosylation sites to be modified. Though the extent of O-glycosylation is low, site T323 has been conserved in all variants. This could highlight the importance of O-glycosylation for viral-host binding.

Herein we have outlined a comprehensive analysis of the N- and O-glycosites and glycoforms present on 6 S proteins (WT, Alpha, Beta, Gamma, Delta, Omicron). This data confirms that all 22 N-glycosylation sites are conserved across the WT, Alpha, Beta, Gamma and Omicron variants, and 21 sites are conserved in the Delta variant.

## Supplementary Information

Supplementary Information.

## Data Availability

LC–MS/MS data files and Byonic search results are available from https://glycopost.glycosmos.org/preview/17096263556346a0cc0f734; Pin—7577.
