# Supplementary material for: Site specific N- and O-glycosylation mapping of the spike proteins of SARS-CoV-2 variants of concern
Source: Sci Rep. 2023 Jun 21;13:10053. doi: 10.1038/s41598-023-33088-0 (PMC10284906; doi:10.1038/s41598-023-33088-0)
Supplement: Supplementary file 1 — Supplementary Information. [file 41598_2023_33088_MOESM1_ESM.docx]

**Supporting information**

Both N- and O- glycosylation sites are highly conserved in SARS-CoV-2 spike proteins across variants of concern, but glycosylation patterns vary across the variants

Asif Shajahan^1^#, Lauren E. Pepi^2^, Bhoj Kumar^2^, Nathan B. Murray^2^, Parastoo Azadi^2^*

^1^Vaccine Production Program, Vaccine Research Center, National Institutes of Health, 9 W Watkins Mill Rd, Gaithersburg, MD 20877; ^2^Complex Carbohydrate Research Center, University of Georgia, Athens, GA 30602

*Correspondence Author, ^#^Co-correspondence Author.

E-mail address: asif.shajahan@nih.gov (Asif Shajahan), lauren.pepi@uga.edu (Lauren E. Pepi), bhoj.Kumar@uga.edu (Bhoj Kumar), nathan.murray@uga.edu (Nathan B. Murray), cheiss@ccrc.uga.edu (Christian Heiss), azadi@ccrc.uga.edu (Parastoo Azadi).

**Table of Contents**

| **Content** | **Figure Number** |
| --- | --- |
| SDS-polyacrylamide gel of spike variants | S1 |
| Experimental Design | S2 |
| Glycoform distribution at site N17 | S3 |
| Glycoform distribution at site N61 | S4 |
| Glycoform distribution at site N74 | S5 |
| Glycoform distribution at site N122 | S6 |
| Glycoform distribution at site N149 | S7 |
| Glycoform distribution at site N165 | S8 |
| Glycoform distribution at site N188 | S9 |
| Glycoform distribution at site N234 | S10 |
| Glycoform distribution at site N282 | S11 |
| Glycoform distribution at site N331 | S12 |
| Glycoform distribution at site N343 | S13 |
| Glycoform distribution at site N603 | S14 |
| Glycoform distribution at site N616 | S15 |
| Glycoform distribution at site N657 | S16 |
| Glycoform distribution at site N709 | S17 |
| Glycoform distribution at site N717 | S18 |
| Glycoform distribution at site N801 | S19 |
| Glycoform distribution at site N1074 | S20 |
| Glycoform distribution at site N1098 | S21 |
| Glycoform distribution at site N1134 | S22 |
| Glycoform distribution at site N1158 | S23 |
| Glycoform distribution at site N1173 | S24 |
| Glycoform distribution at site N1194 | S25 |
| Glycoform distribution at site T323 | S26 |
| PCA and heat map of site N17 and N61 | S27 |
| PCA and heat map of site N74 and N122 | S28 |
| PCA and heat map of site N149 and N165 | S29 |
| PCA and heat map of site N234 and N282 | S30 |
| PCA and heat map of site N331 and N343 | S31 |
| PCA and heat map of site N603 and N616 | S32 |
| PCA and heat map of site N657 and N709 | S33 |
| PCA and heat map of site N717 and N801 | S34 |
| PCA and heat map of site N1074 and N1098 | S35 |
| PCA and heat map of site N1134 and N1158 | S36 |
| PCA and heat map of site N1173 and N1194 | S37 |
| Distribution of N-glycan types across the variants – determined by N-glycomics | S38 |
| ESI-MS spectrum of N-glycans – WT spike protein. | S39 |
| ESI-MS spectrum of N-glycans - alpha variant spike protein. | S40 |
| ESI-MS spectrum of N-glycans - beta variant spike protein. | S41 |
| ESI-MS spectrum of N-glycans - gamma variant spike protein. | S42 |
| ESI-MS spectrum of N-glycans - delta variant spike protein. | S43 |
| ESI-MS spectrum of N-glycans - omicron variant spike protein. | S44 |
| Sialic Acid Linkage Analysis | S45 |
| Sialic linkages on N-glycans - spike protein of variants | Table S1 |
| LC solvent gradient for glycoproteomics | Table S2 |
| LC solvent gradient for glycomics | Table S3 |


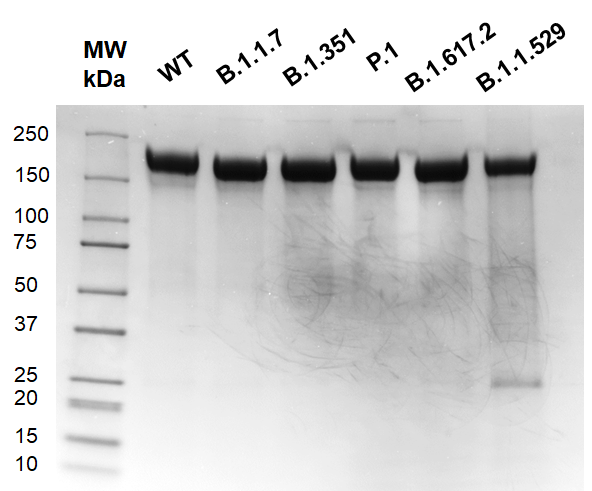


Figure S 1: Coomassie stained SDS-polyacrylamide gel of recombinantly expressed spike proteins of SARS-CoV-2 Wuhan-Hu-1 and VOCs (Wuhan-Hu-1/WT, VOCs- alpha/B.1.1.7, beta/B.1.351, gamma/P.1, delta/B.1.617.2, and omicron B.1.1.529) expressed under identical conditions showing similar purity and migration profile.


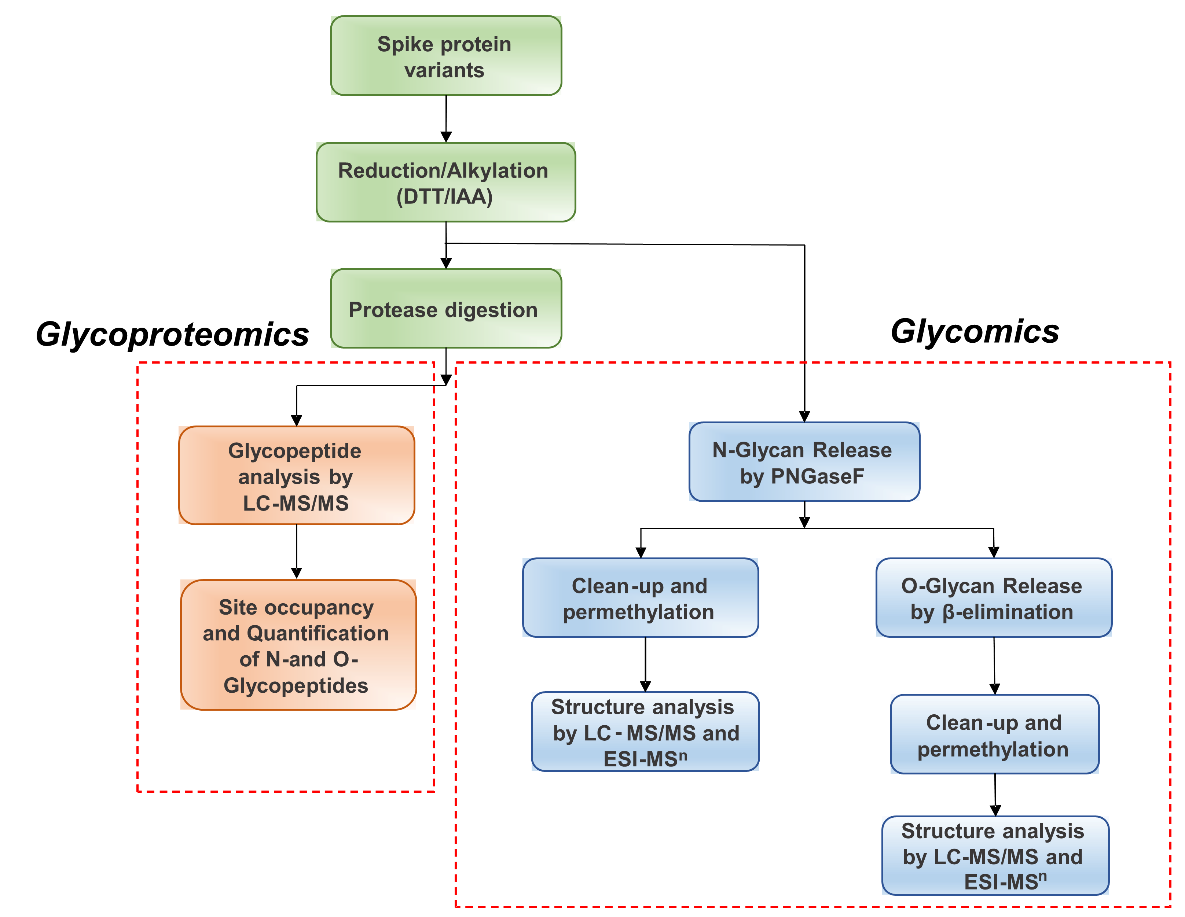


Figure S 2: Glycoproteomics and glycomics workflow followed for the quantitative and qualitative profiling of N-and O-glycosylation on the S protein of SARS-CoV-2 Wuhan-Hu-1 and VOCs.

Figure S 3: Relative abundances of N-glycans at site N17 across the variants. N20 in the case of P.1 (Gamma variant).

Figure S 4: Relative abundances of N-glycans at site N61 across the variants.

Figure S 5: Relative abundances of N-glycans at site N74 across the variants.

Figure S 6: Relative abundances of N-glycans at site N122 across the variants.

Figure S 7: Relative abundances of N-glycans at site N149 across the variants.

Figure S 8: Relative abundances of N-glycans at site N165 across the variants.

Figure S 9. Relative abundances of N-glycans at site N188 in P.1 sample. N188 site is absent in all other variants.

Figure S 10: Relative abundances of N-glycans at site N234 across the variants.

Figure S 11: Relative abundances of N-glycans at site N282 across the variants.

Figure S 12: Relative abundances of N-glycans at site N331 across the variants.

Figure S 13: Relative abundances of N-glycans at site N343 across the variants.

Figure S 14: Relative abundances of N-glycans at site N603 across the variants.

Figure S 15: Relative abundances of N-glycans at site N616 across the variants.

Figure S 16: Relative abundances of N-glycans at site N657 across the variants.

Figure S 17: Relative abundances of N-glycans at site N709 across the variants.

Figure S 18: Relative abundances of N-glycans at site N717 across the variants.

Figure S 19: Relative abundances of N-glycans at site N801 across the variants.

Figure S 20: Relative abundances of N-glycans at site N1074 across the variants.

Figure S 21: Relative abundances of N-glycans at site N1098 across the variants.

Figure S 22: Relative abundances of N-glycans at site N1134 across the variants.

Figure S 23: Relative abundances of N-glycans at site N1158 across the variants.

Figure S 24: Relative abundances of N-glycans at site N1173 across the variants.

Figure S 25: Relative abundances of N-glycans at site N1194 across the variants.

Figure S 26: Relative abundances of O-glycans at site T323 across the variants.


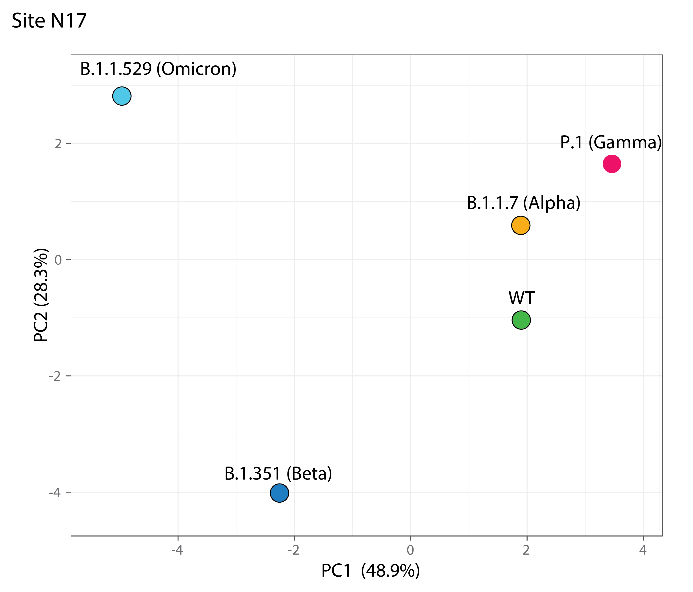

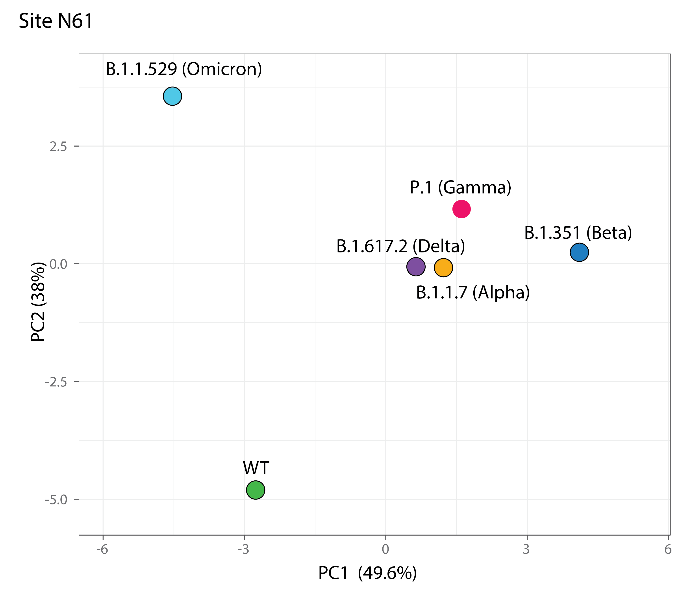

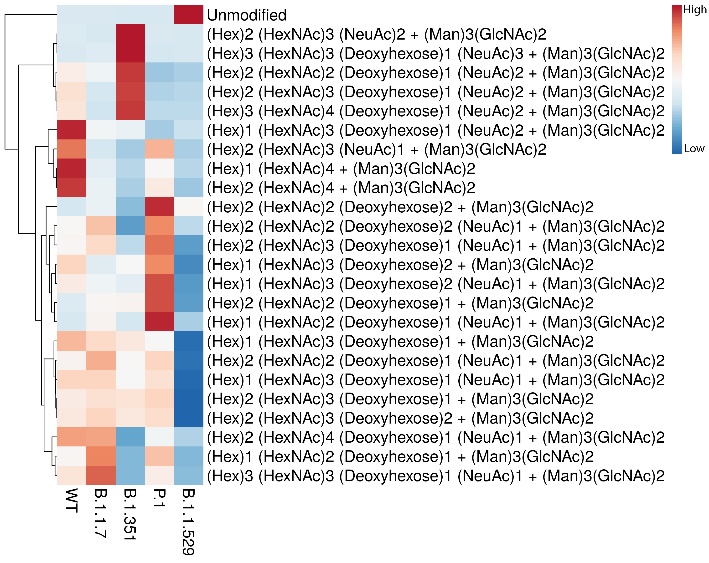

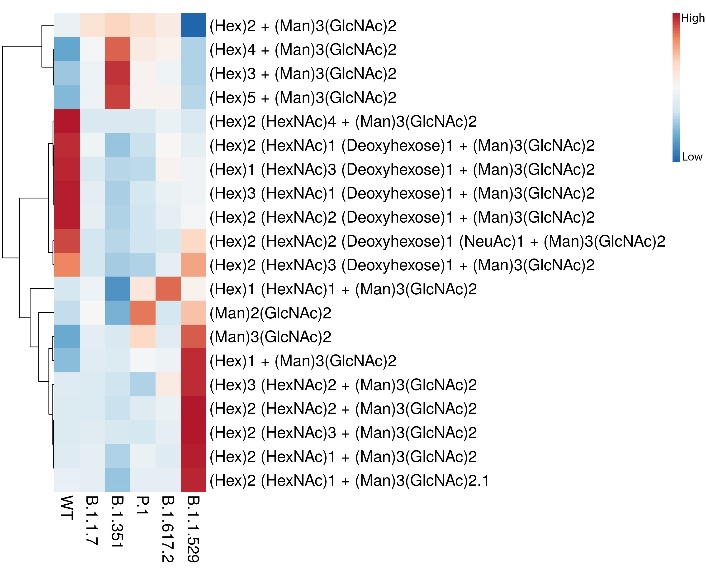


Figure S 27: PCA and Heat map comparison of N-glycans at site N17 (N20 in the case of P.1, Gamma) and N61 of variants.


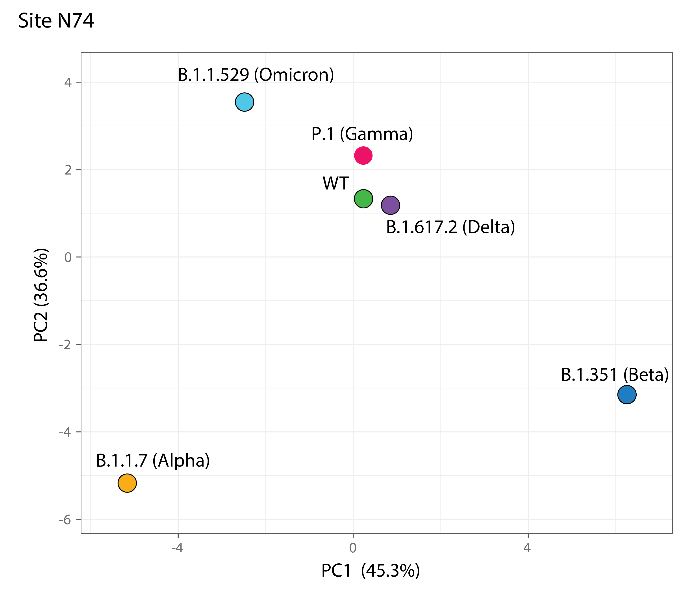

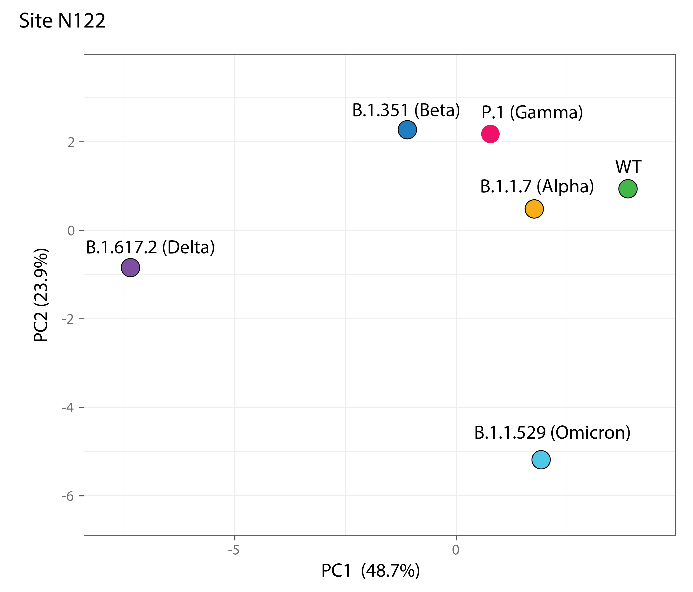

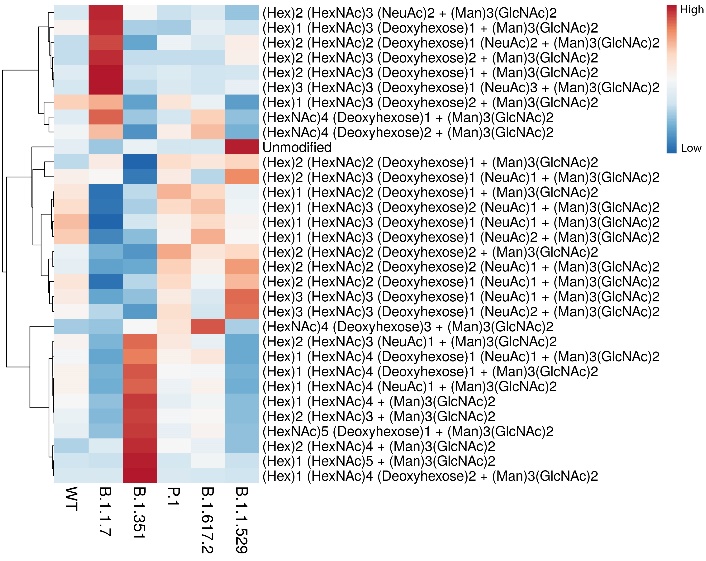

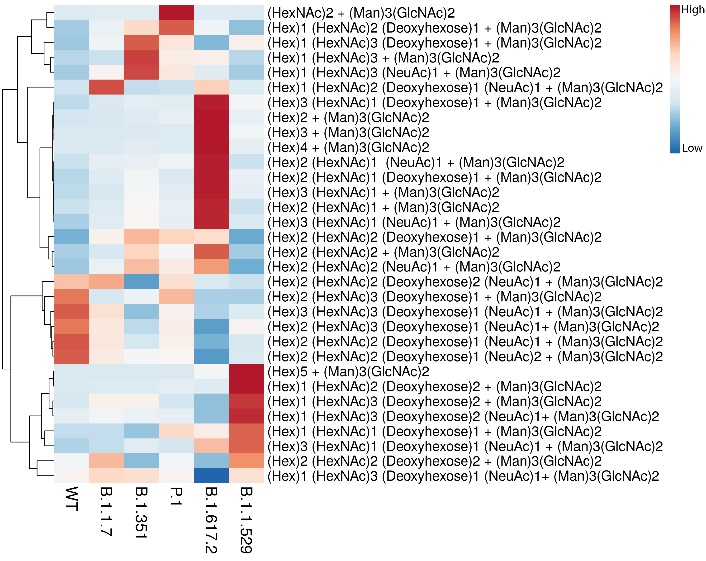


Figure S 28: PCA and Heat map comparison of N-glycans at site N74 and N122 of variants.


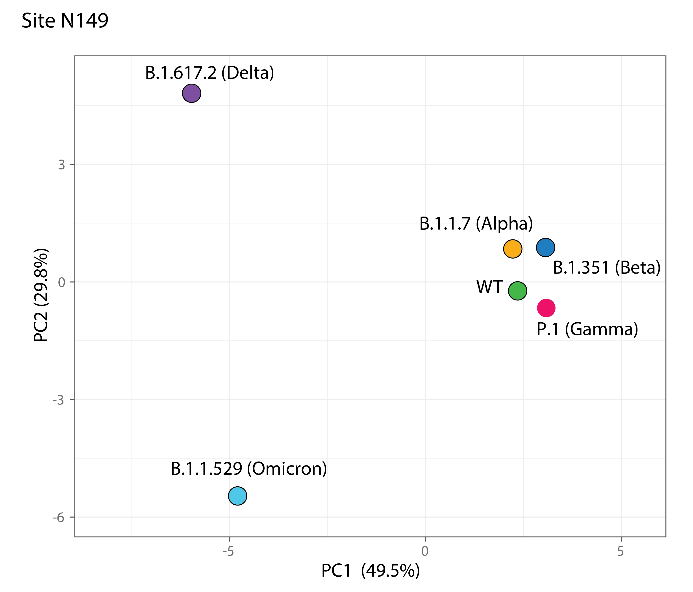

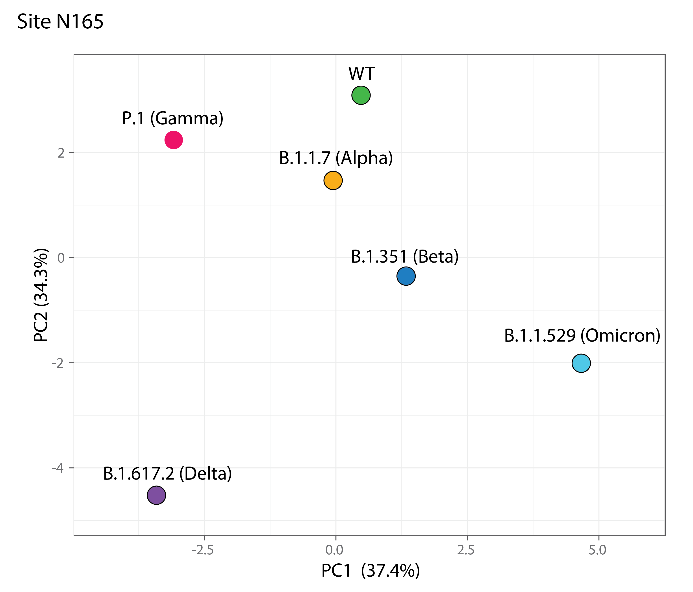

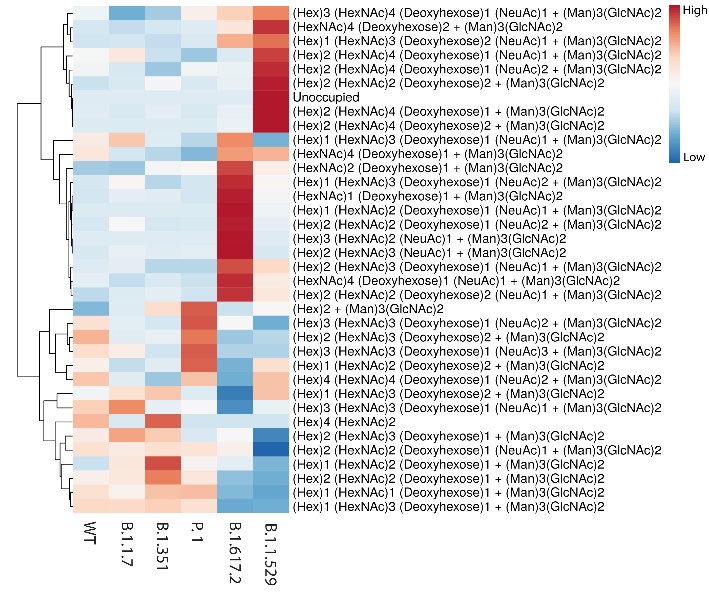

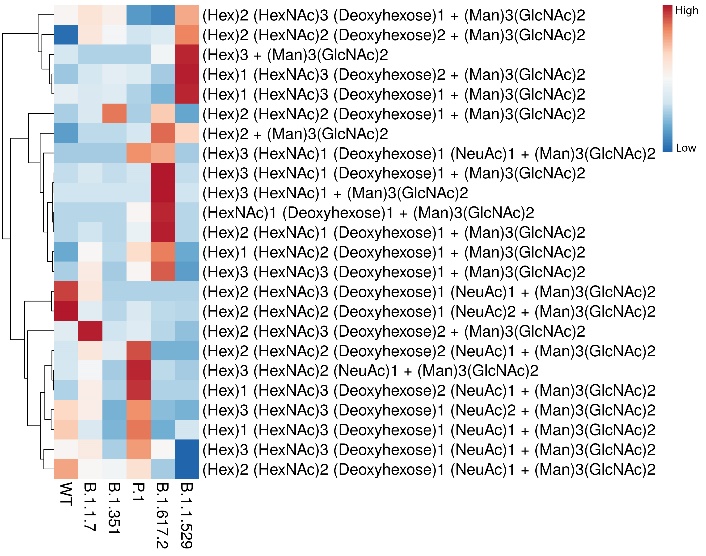


Figure S 29: PCA and Heat map comparison of N-glycans at site N149 and N165 of variants.


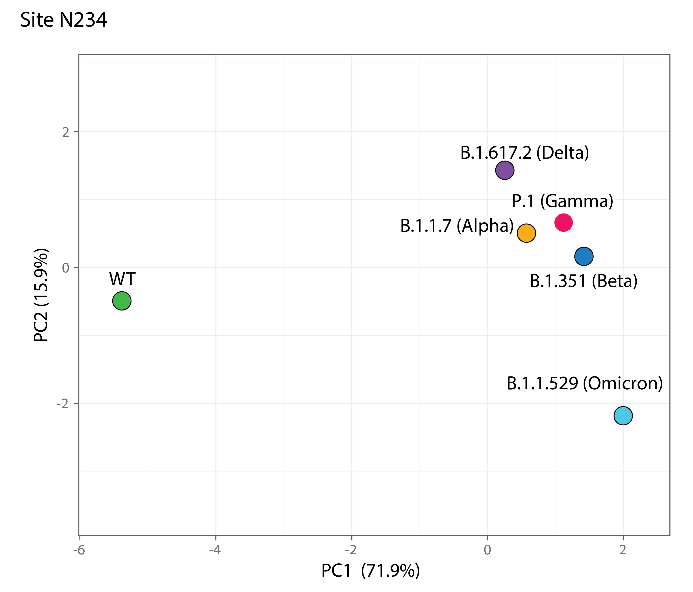

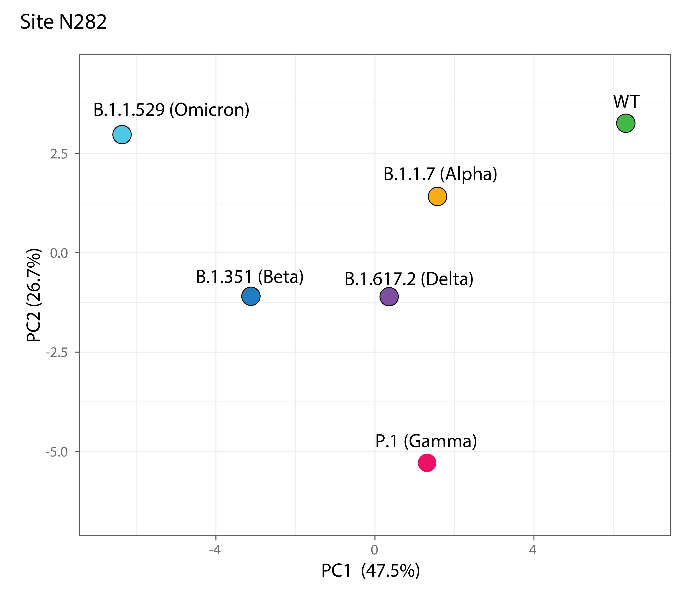

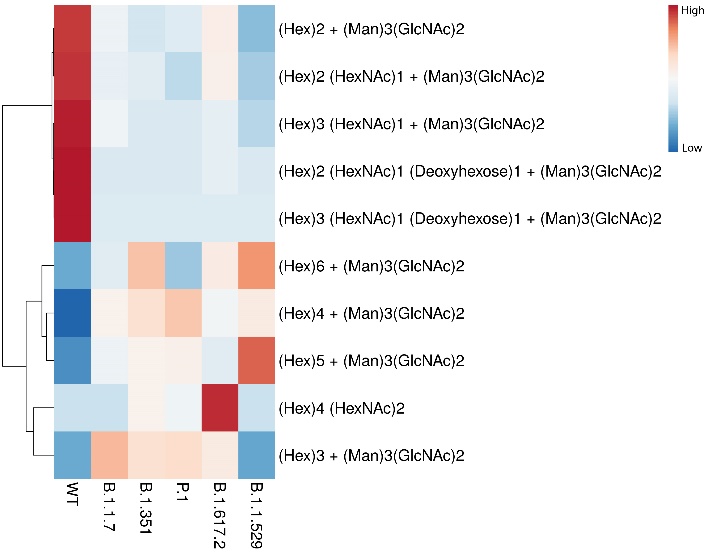

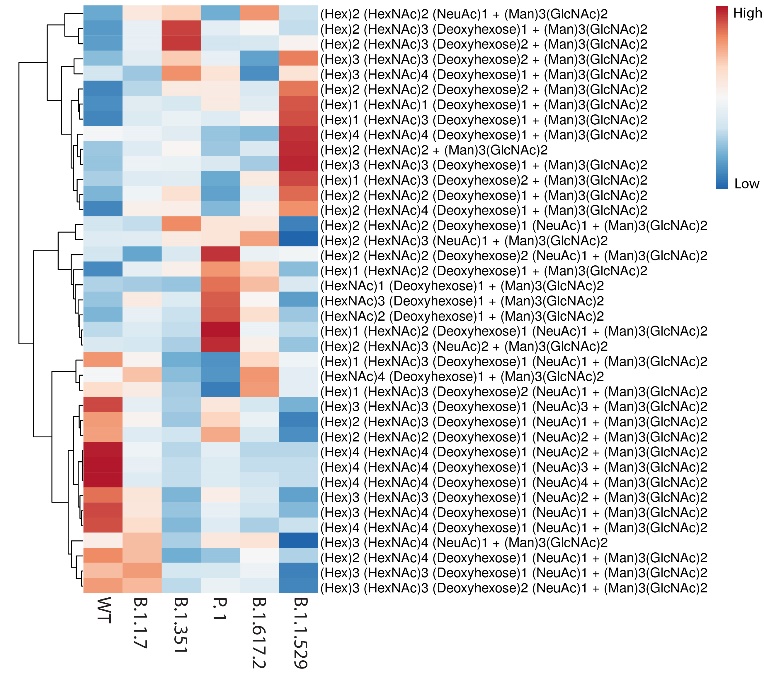


Figure S 30: PCA and Heat map comparison of N-glycans at site N234 and N282 of variants.


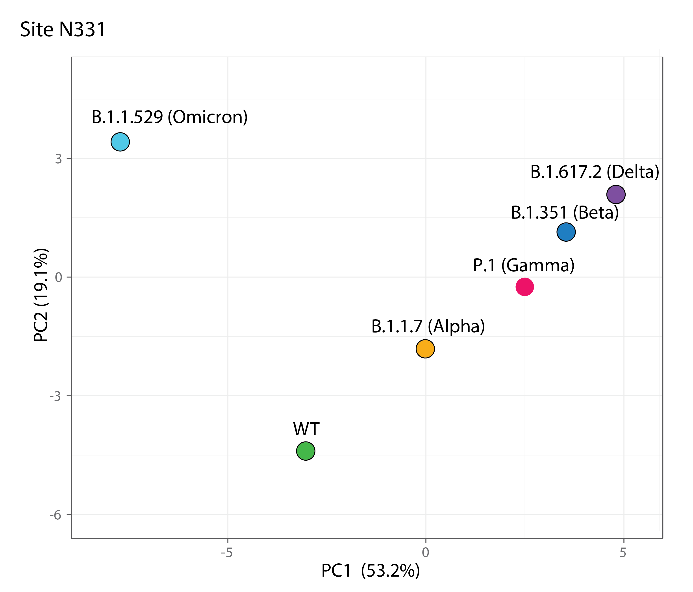

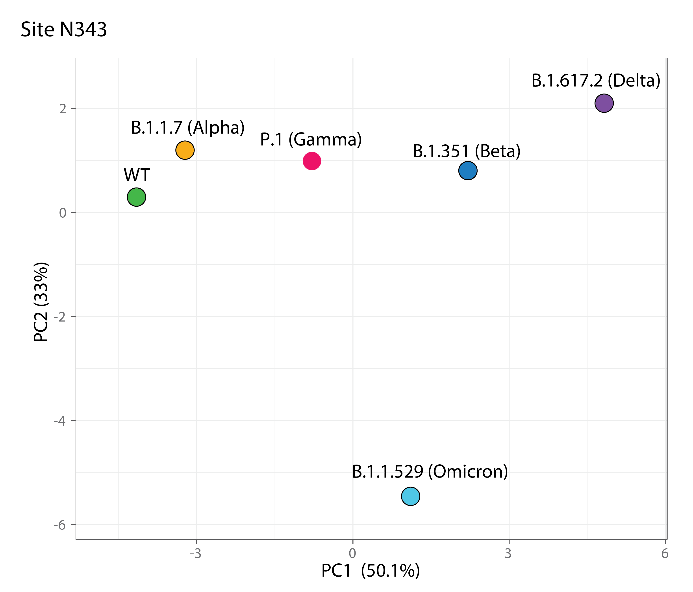

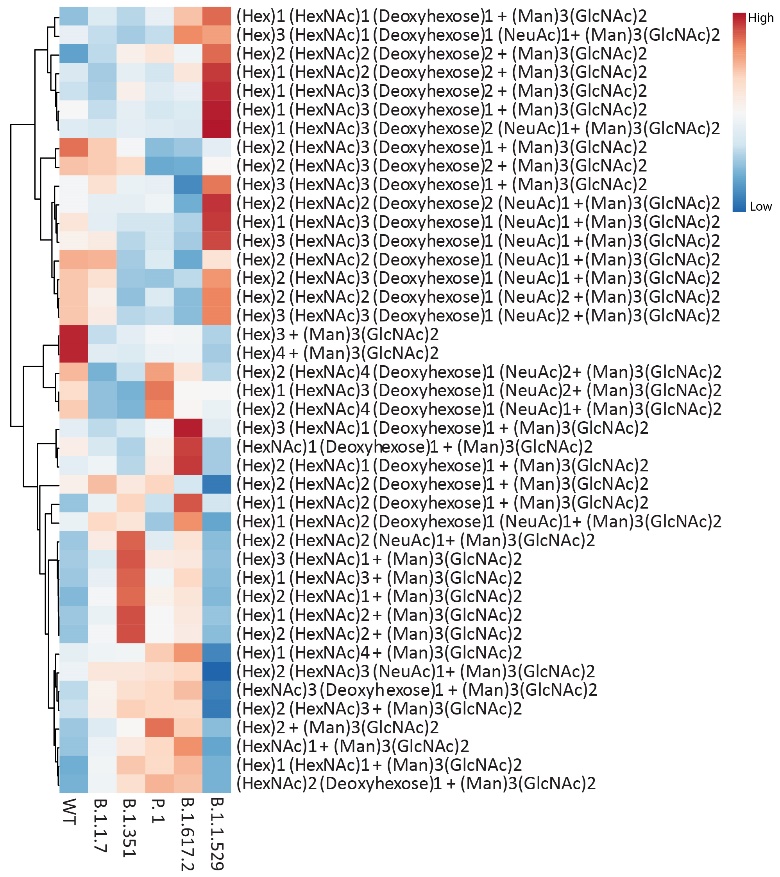

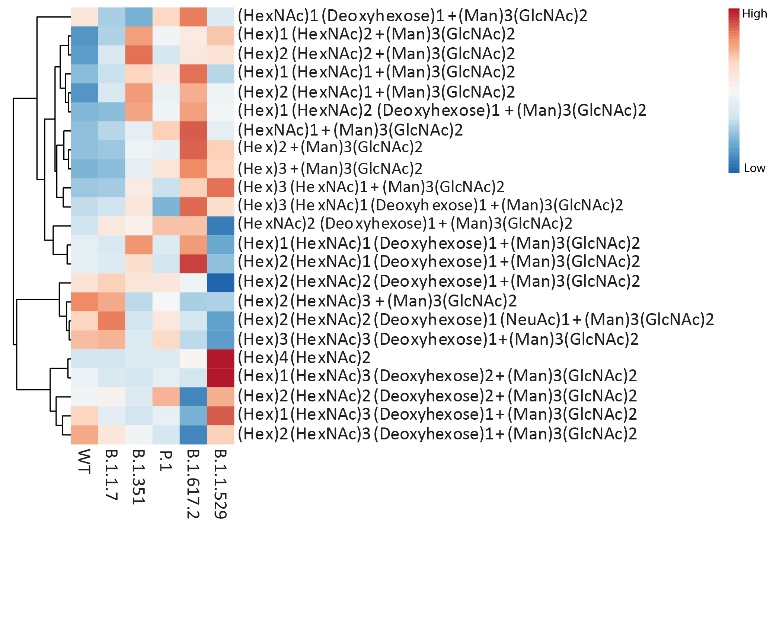


Figure S 31: PCA and Heat map comparison of N-glycans at site N331 and N343 of variants.


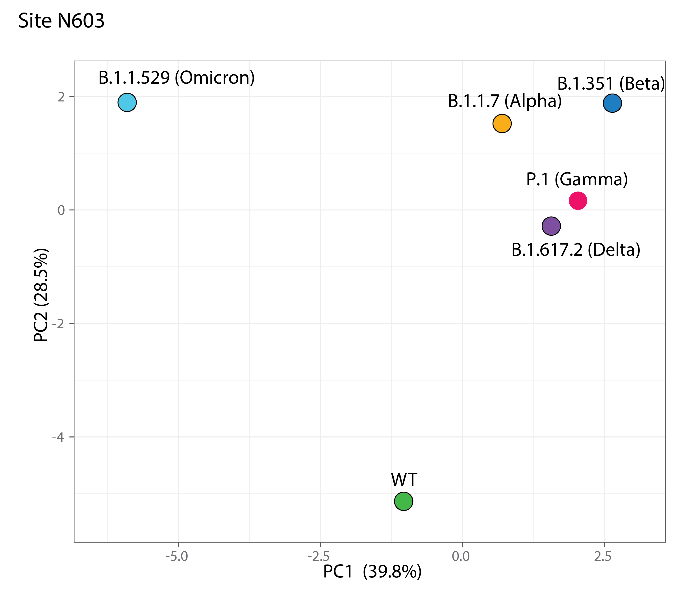

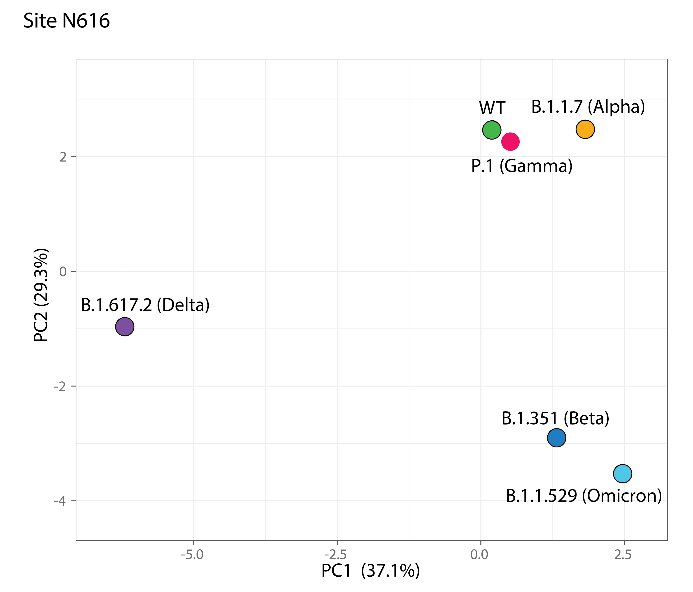

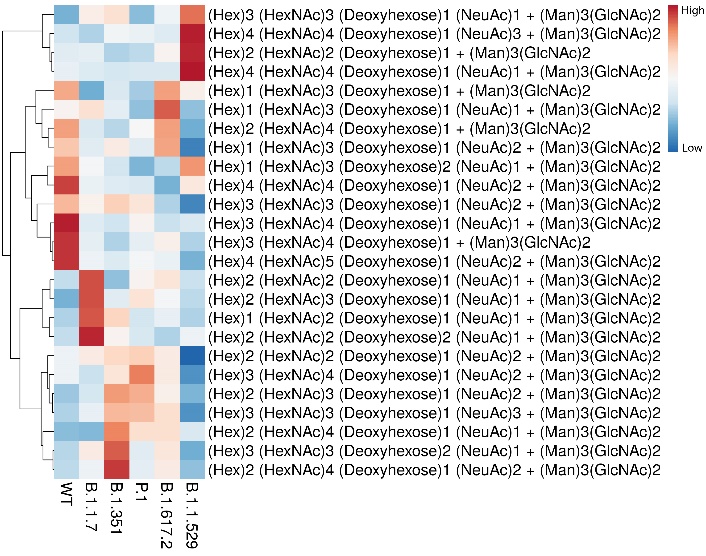

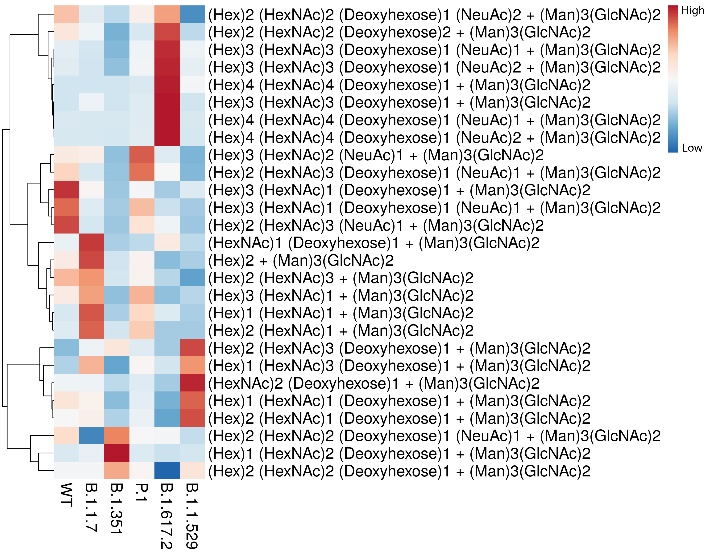


Figure S 32: PCA and Heat map comparison of N-glycans at site N603 and N616 of variants.


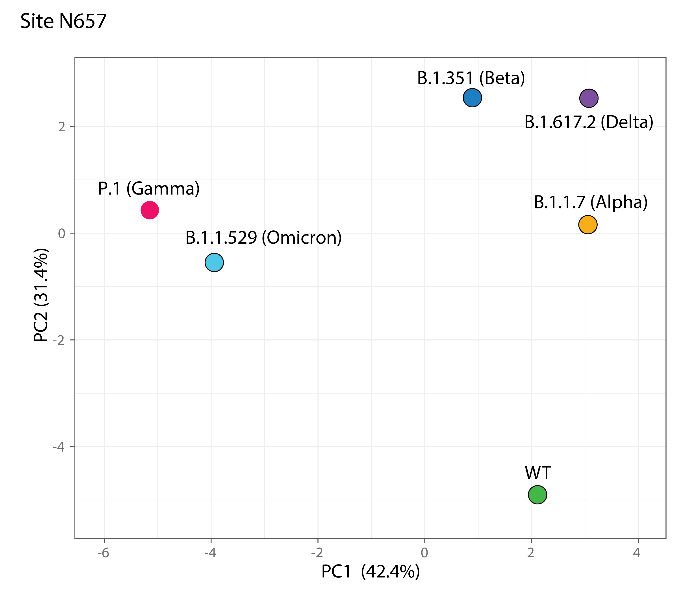

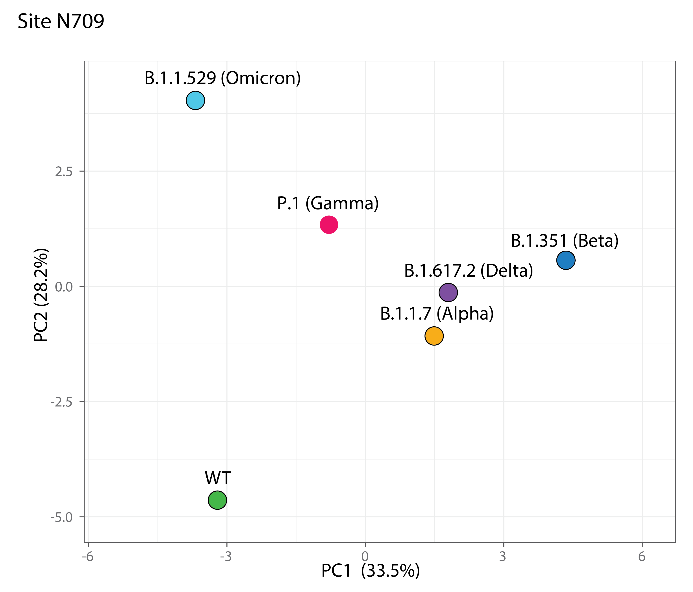

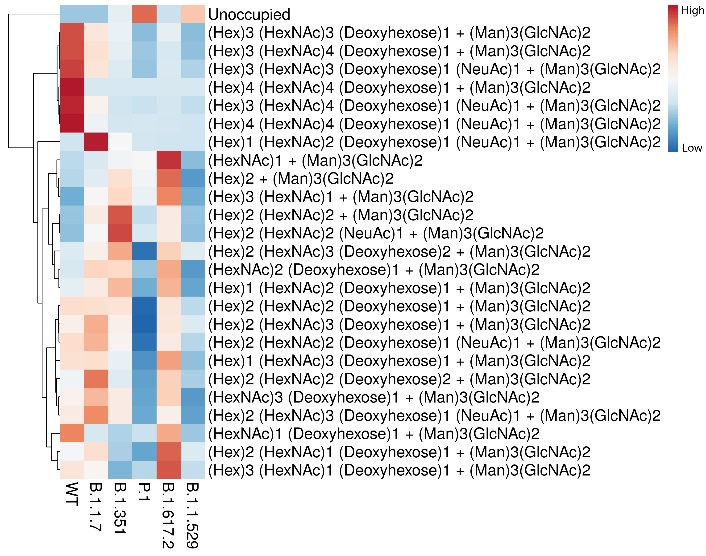

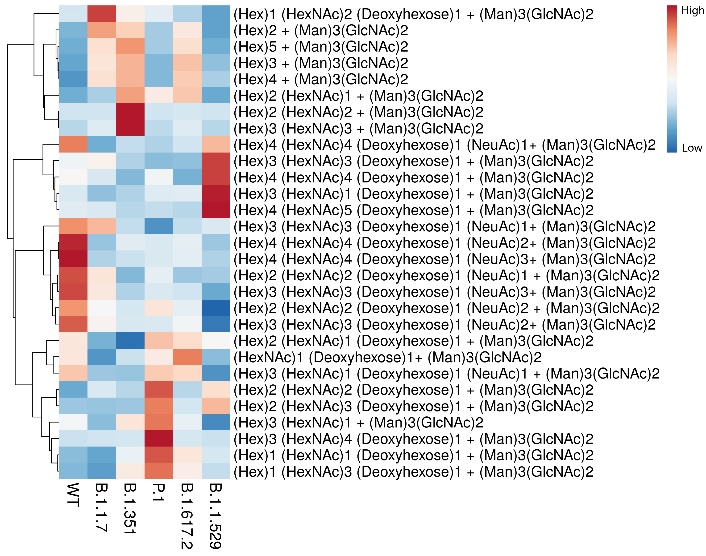


Figure S 33: PCA and Heat map comparison of N-glycans at site N657 and N709 of variants.


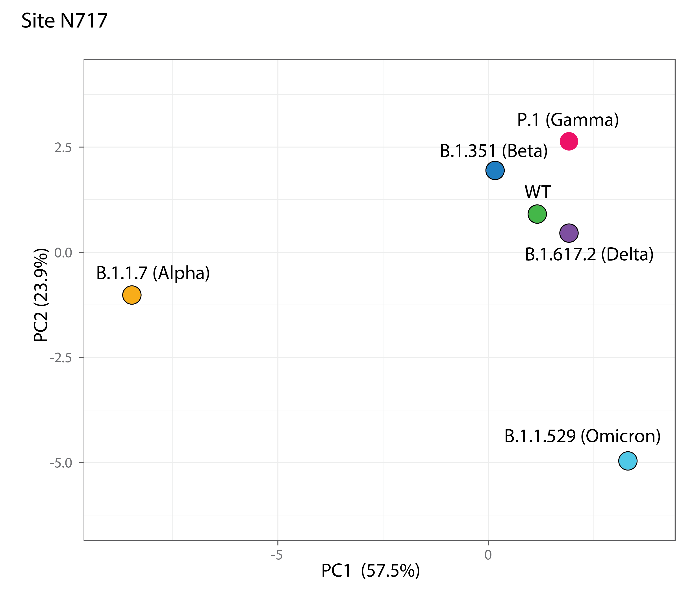

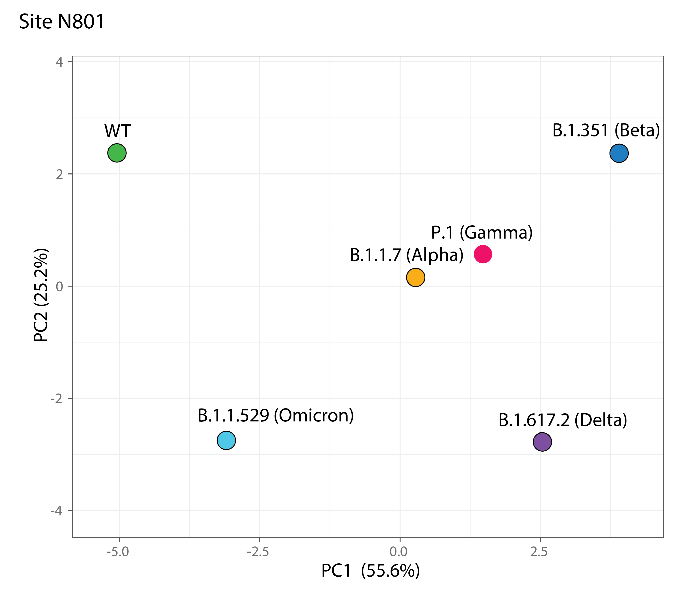

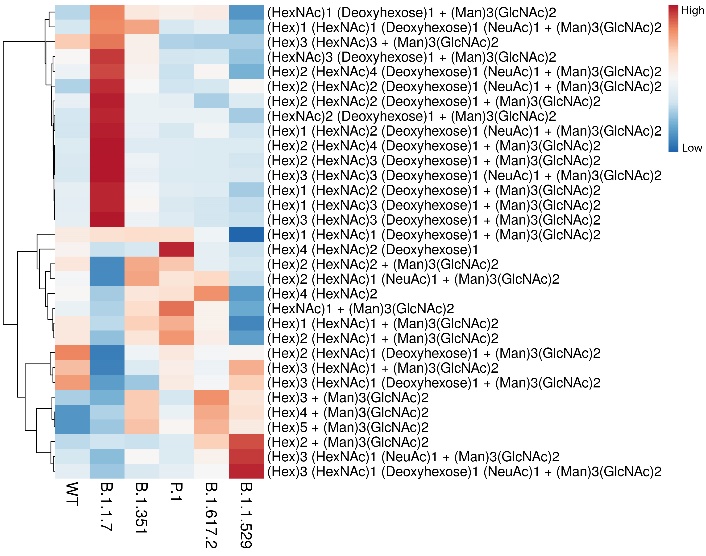

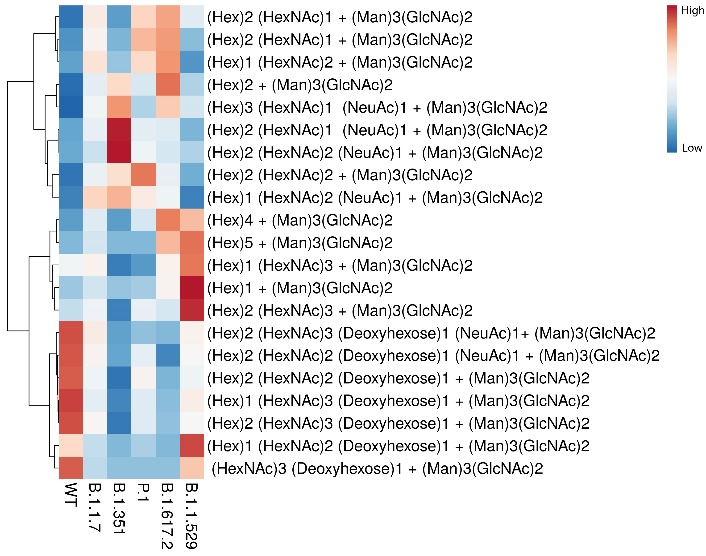


Figure S 34: PCA and Heat map comparison of N-glycans at site N717 and N801 of variants.


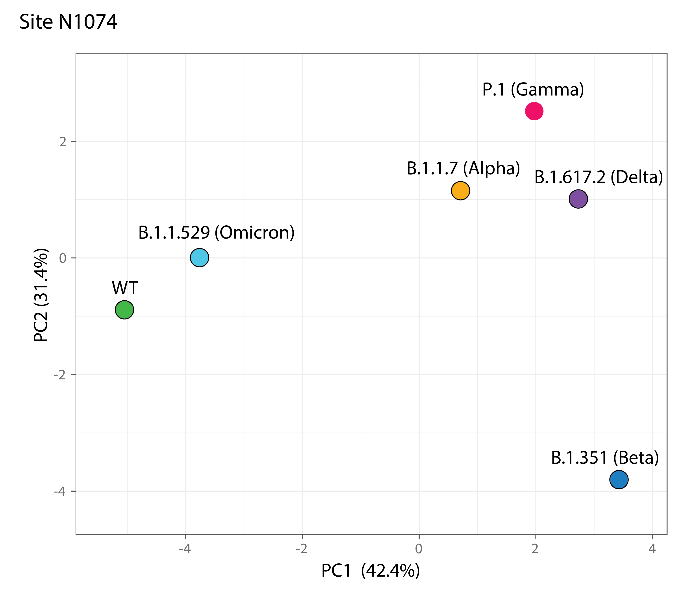

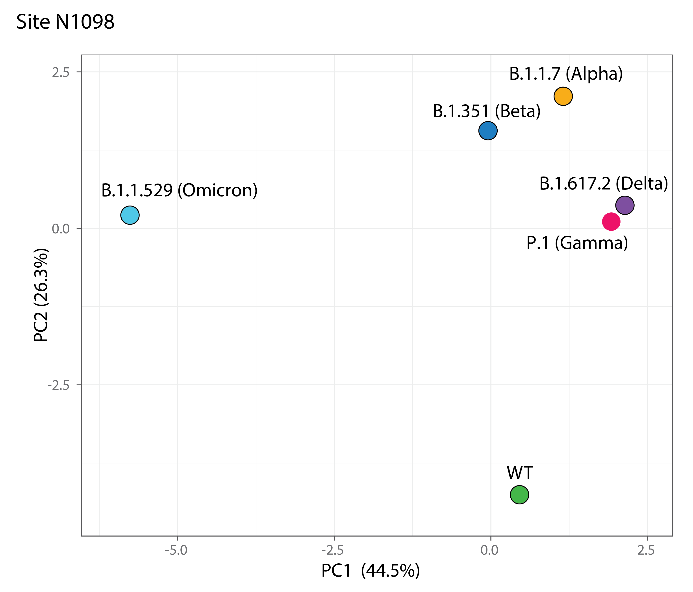

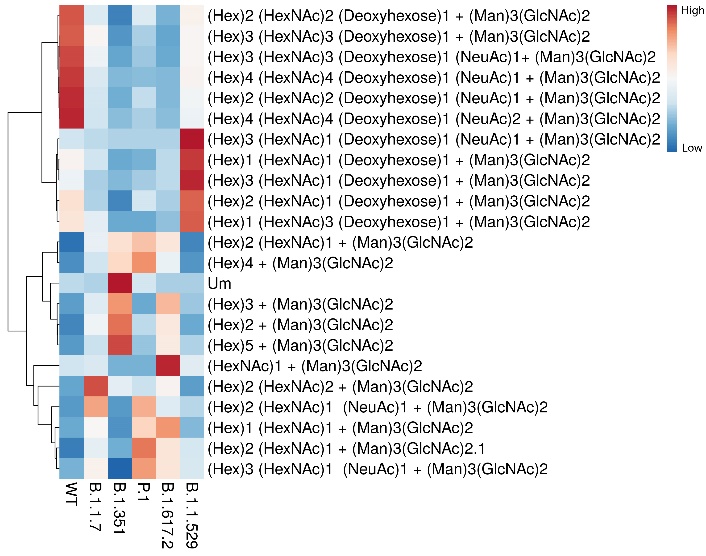

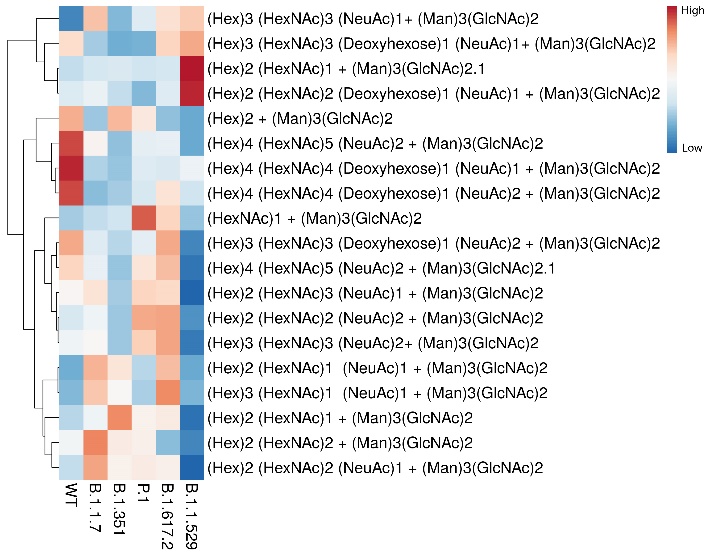


Figure S 35: PCA and Heat map comparison of N-glycans at site N1074 and N1098 of variants.


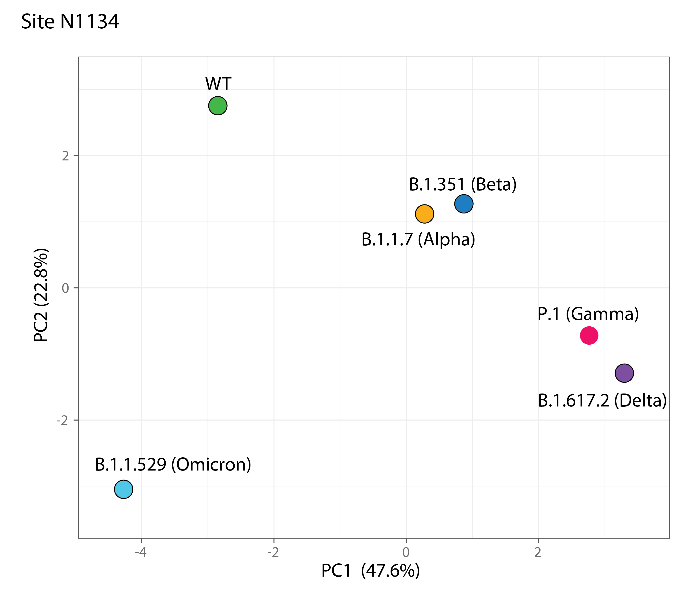

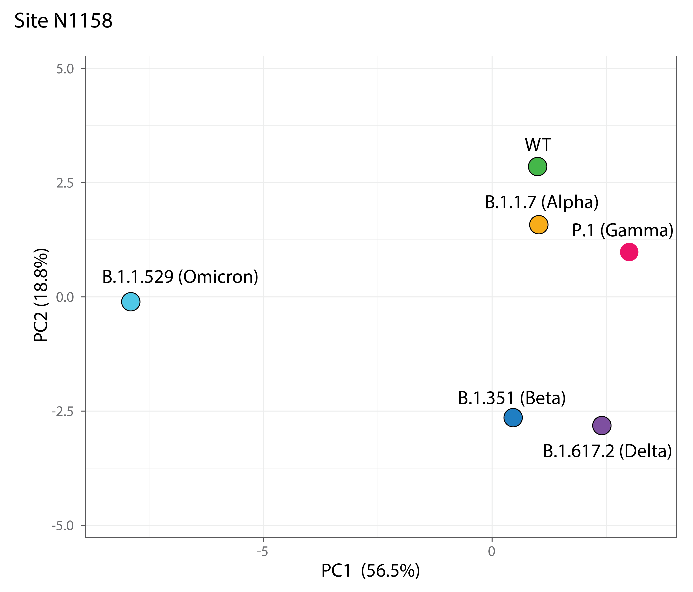

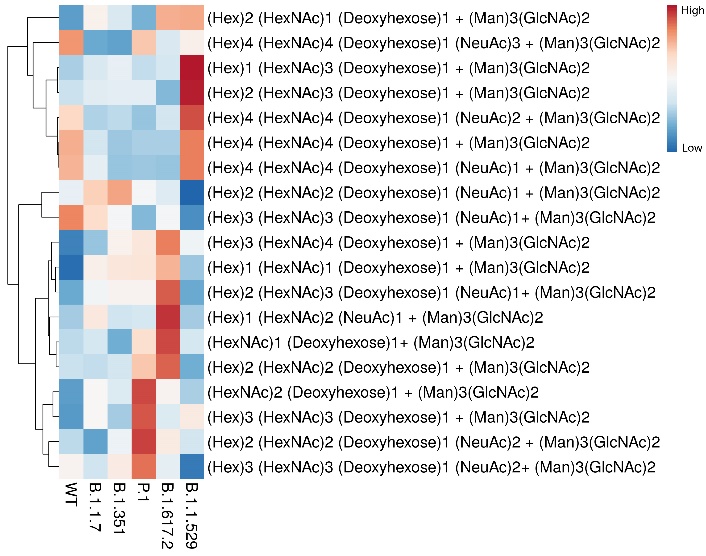

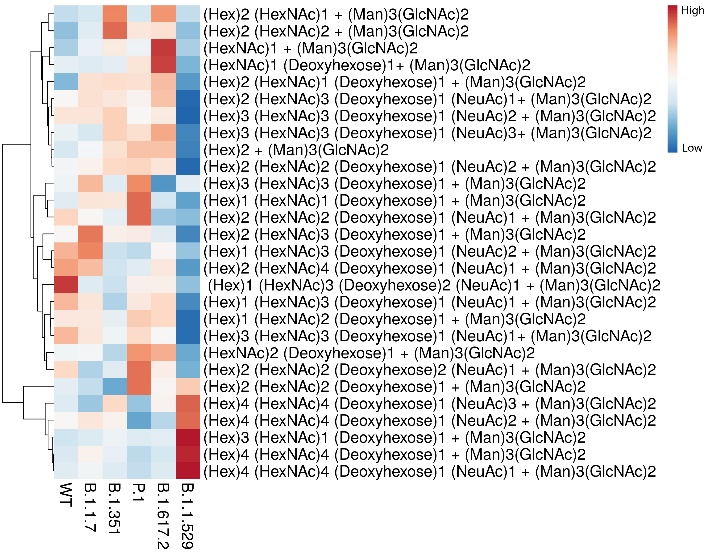


Figure S 36: PCA and Heat map comparison of N-glycans at site N1134 and N1158 of variants.


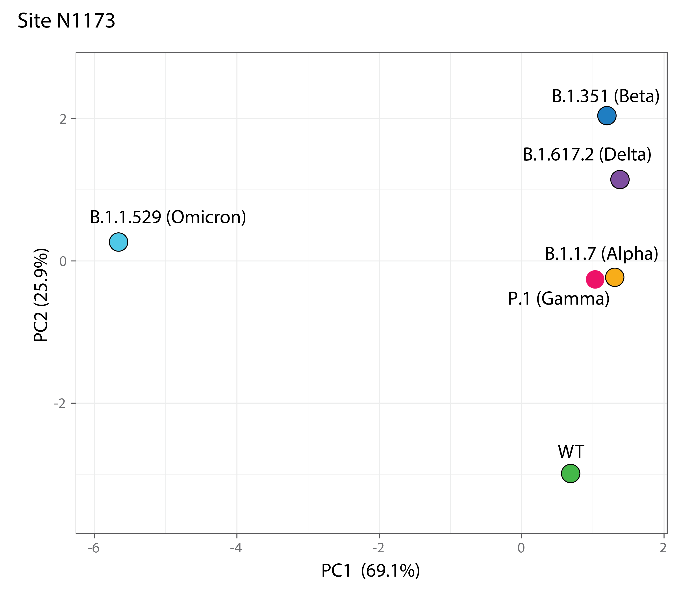

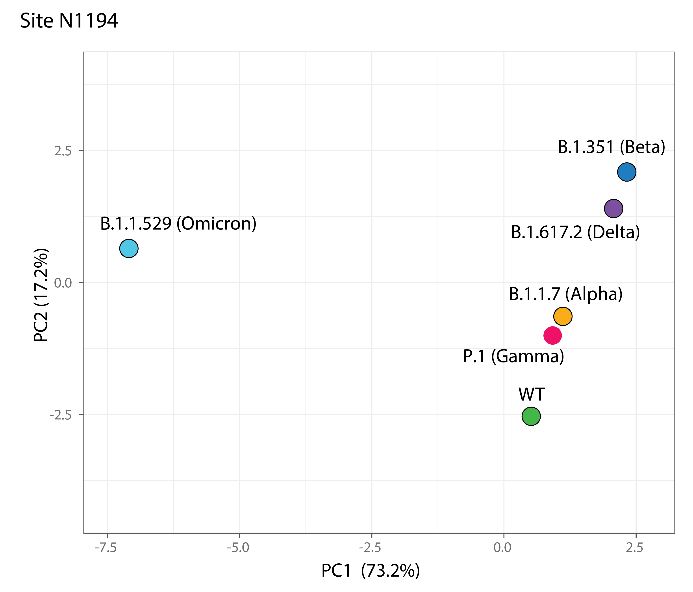

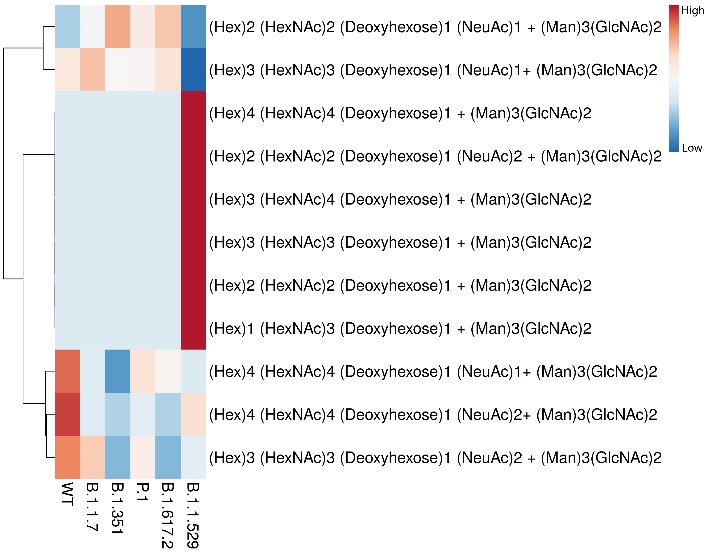

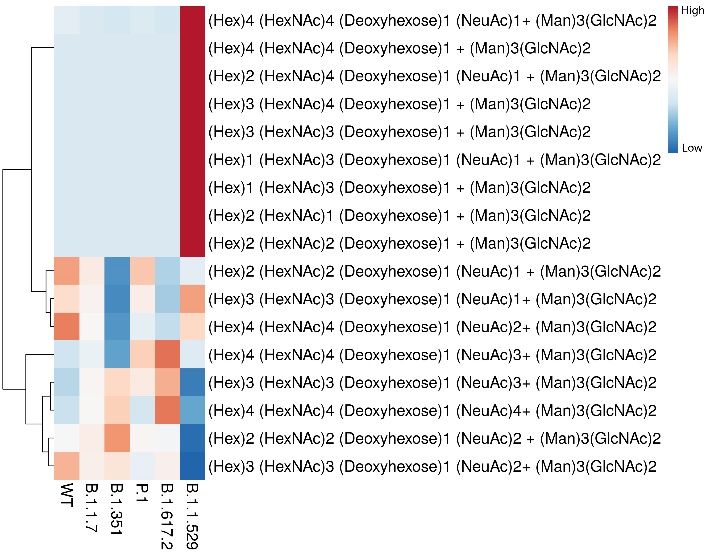


Figure S 37: PCA and Heat map comparison of N-glycans at site N1173 and N1194 of variants.

Figure S 38: Distribution of N-glycan types across the variants based on N-glycomics results. Data was collected in duplicate, and error bars represent the standard deviation.


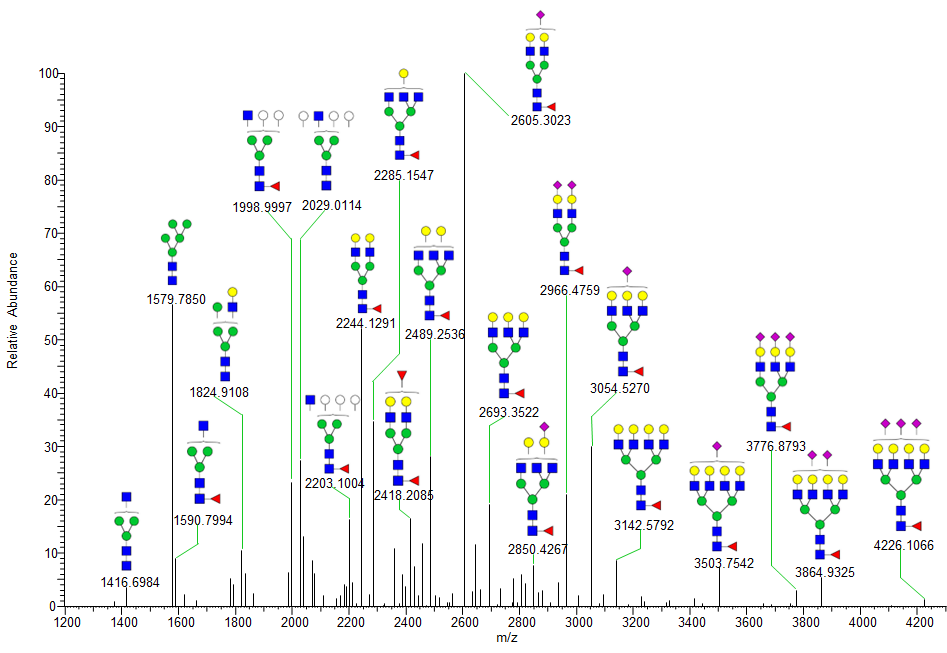


Figure S 39: Deconvoluted ESI-MS spectrum of permethylated N-glycans released from WT (Wuhan-Hu-1) spike protein. Spectrum is obtained by averaging 72 min LC-MS run.


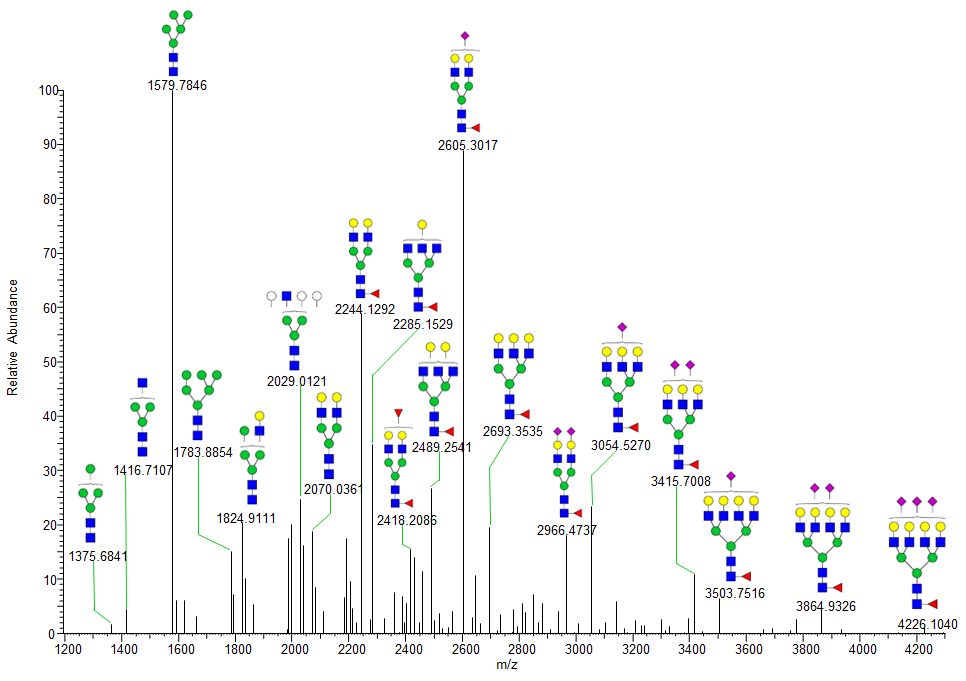


Figure S 40: Deconvoluted ESI-MS spectrum of permethylated N-glycans released from alpha (B.1.1.7) variant spike protein. Spectrum is obtained by averaging 72 min LC-MS run.


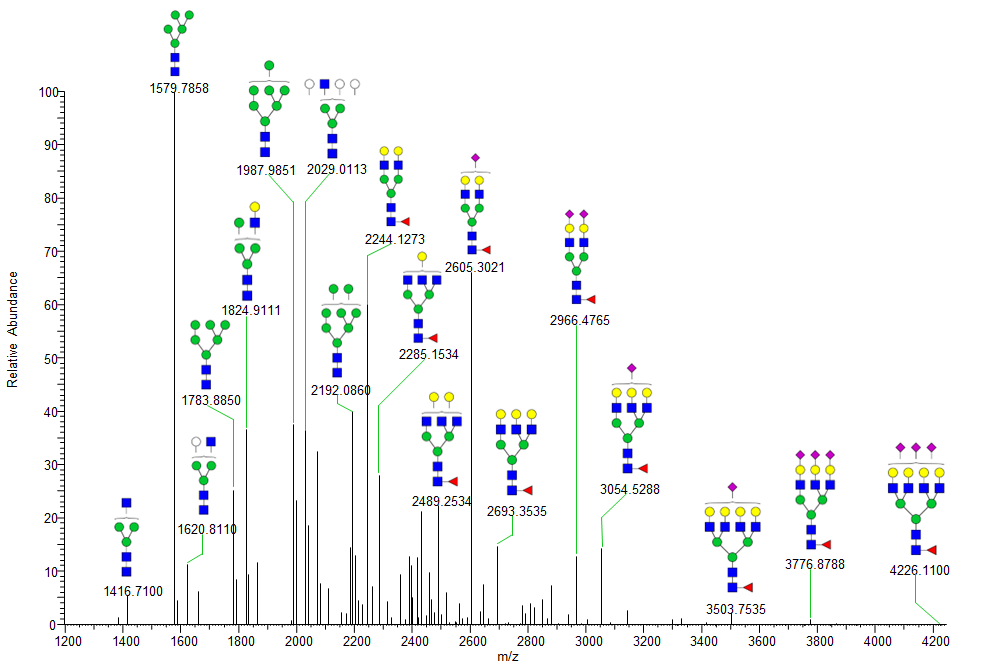


Figure S 41: Deconvoluted ESI-MS spectrum of permethylated N-glycans released from beta (B.1.351) variant spike protein. Spectrum is obtained by averaging 72 min LC-MS run.


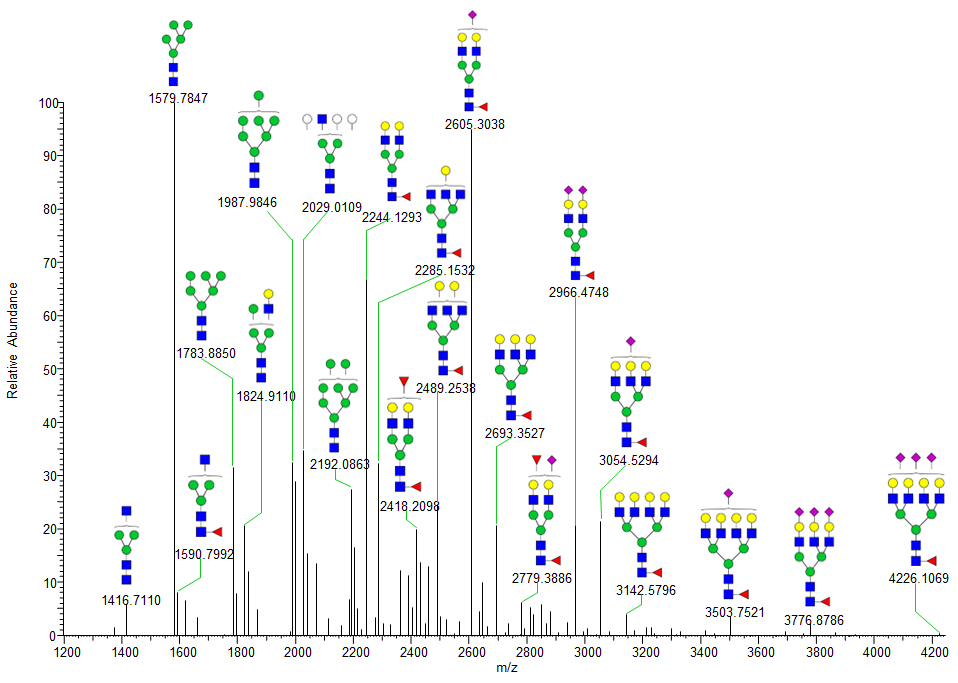


Figure S 42: Deconvoluted ESI-MS spectrum of permethylated N-glycans released from gamma (P.1) variant spike protein. Spectrum is obtained by averaging 72 min LC-MS run.


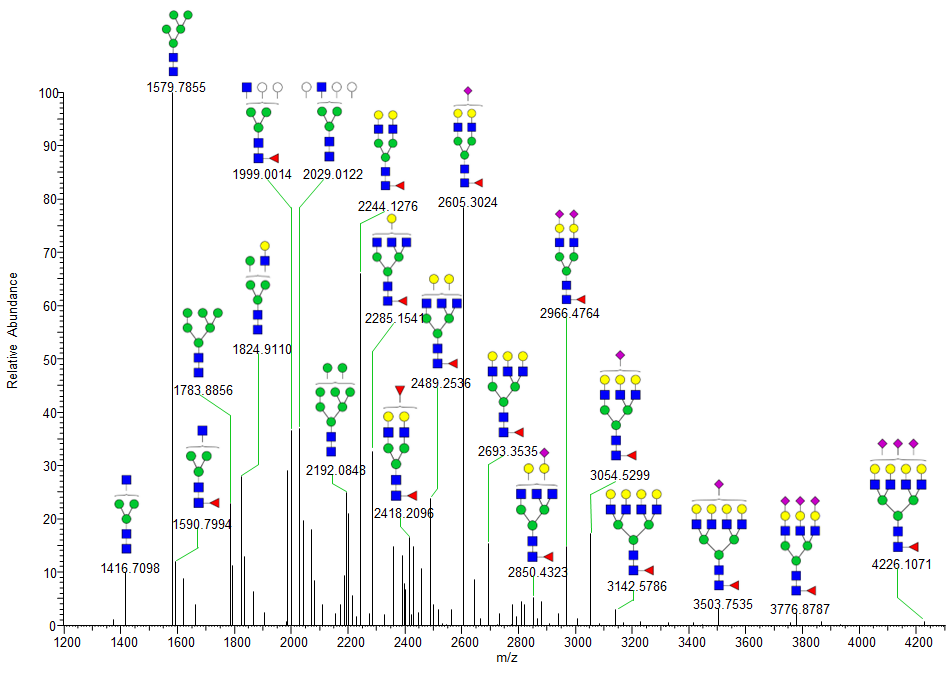


Figure S 43: Deconvoluted ESI-MS spectrum of permethylated N-glycans released from delta (B.1.617.2) variant spike protein. Spectrum is obtained by averaging 72 min LC-MS run.


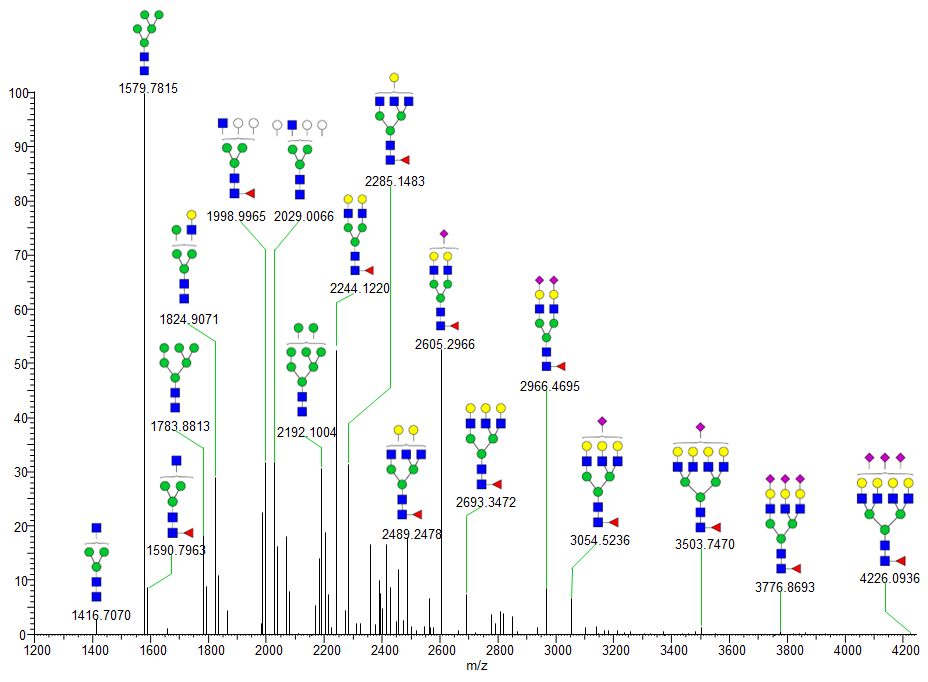


Figure S 44: Deconvoluted ESI-MS spectrum of permethylated N-glycans released from omicron (B.1.1.529) variant spike protein. Spectrum is obtained by averaging 72 min LC-MS run.


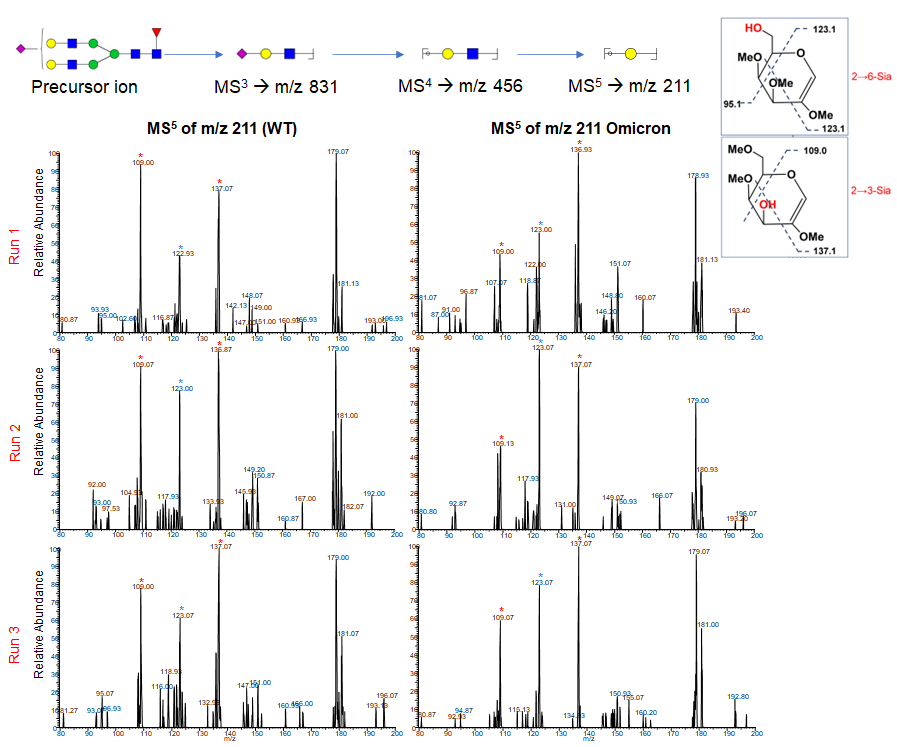


Figure S 45: Sialic acid linkage comparison on N-glycans released from Wuhan-Hu-1 (WT) SARS-CoV-2 and Omicron by ESI-MS^n^ of permethylated glycans (M+Li) showed the presence of both 2,3 and 2,6 linked sialic acid in both WT and Omicron but slightly increased levels of 2,6 linked sialic acid in Omicron variant. Fragment ions at m/z 109 (*) and 137 (*) are diagnostic for 2,3 linkage. Fragment ion at m/z 123 (*) is diagnostic for 2,6 linkage.

*Table S1: Sialic linkage observed across the N-glycans released from spike protein of variants – determined by ESI-MSn*

| **Structure (Precursor)** | **WT** | B.1.1.7 | **B.1.351** | **P.1** | **B.1.617.2** | **B.1.1.529** |
| --- | --- | --- | --- | --- | --- | --- |
| 2390 (m/z 796, z=3) | Not detected | Not detected | Not detected | Not detected | Not detected | α2,3 > α2,6 |
| 2431 (m/z 809, z=3) | α2,3 > α2,6 | Not detected | α2,3 > α2,6 | α2,3 > α2,6 | α2,3 > α2,6 | α2,3 = α2,6 |
| 2605 (m/z 867, z=3) | α2,3 > α2,6 | α2,3 > α2,6 | α2,3 > α2,6 | α2,3 > α2,6 | α2,3 > α2,6 | α2,3 = α2,6 |
| 2646 (m/z 881, z=3) | Not detected | α2,3 > α2,6 | Not detected | Not detected | Not detected | Not detected |
| 2779 (m/z 925, z=3) | Not detected | Not detected | Not detected | Not detected | Not detected | α2,3 > α2,6 |
| 2880 (m/z 959, z=3) | Not detected | Not detected | α2,3 > α2,6 | Not detected | Not detected | Not detected |
| 2966 (m/z 988, z=3) | α2,3 > α2,6 | α2,3 > α2,6 | α2,3 > α2,6 | α2,3 > α2,6 | α2,3 = α2,6 | α2,3 = α2,6 |
| 3054 (m/z 1017, z=3) | α2,3 > α2,6 | α2,3 > α2,6 | α2,3 > α2,6 | α2,3 = α2,6 | α2,3 > α2,6 | Not detected |
| 3415 (m/z 1137, z=3) | α2,3 = α2,6 | α2,3 > α2,6 | Not detected | α2,3 > α2,6 | α2,3 > α2,6 | Not detected |

Table S2. LC solvent gradient used for glycoproteomics data. Solvent A is 0.1% formic acid, and solvent B is 80% acetonitrile with 0.1% formic acid.

| Time | Flow (uL/ min) | %B | Curve |
| --- | --- | --- | --- |
| 0.00 | 0.300 | 4.0 | 5 |
| 3.00 | 0.300 | 4.0 | 5 |
| 155.00 | 0.300 | 40.0 | 5 |
| 156.00 | 0.300 | 90.0 | 5 |
| 167.00 | 0.300 | 90.0 | 5 |
| 167.50 | 0.300 | 4.0 | 5 |
| 180.00 | 0.300 | 4.0 | 5 |

Table S3. LC solvent gradient used for glycomics data. Solvent A is 2% acetonitrile and 0.1% formic acid with 1 mM NaOAc, and solvent B is 80% acetonitrile with 0.1% formic acid and 1 mM NaOAc.

| Time | Flow (uL/ min) | %B | Curve |
| --- | --- | --- | --- |
| 0.00 | 0.300 | 5.0 | 5 |
| 3.00 | 0.300 | 40.0 | 5 |
| 30.00 | 0.300 | 60.0 | 5 |
| 50.00 | 0.300 | 85.0 | 5 |
| 60.00 | 0.300 | 99.0 | 5 |
| 65.00 | 0.300 | 99.0 | 5 |
| 68.00 | 0.300 | 20.0 | 5 |
| 72.00 | 0.300 | 5.0 | 5 |
